# The inhibition of pathological IgE in allergic diseases by natural compounds

**DOI:** 10.1007/s13659-026-00596-1

**Published:** 2026-07-13

**Authors:** Michelle Carnazza, Morgan Begley, Raj K. Tiwari, Jan Geliebter, Nan Yang, Jixun Zhan, Xiu-Min Li

**Affiliations:** 1 Division of Research and Development, General Nutraceutical Technology, LLC, Elmsford, NY USA; 2 Department of Pathology, Microbiology and Immunology, New York Medical College, Valhalla, NY USA; 3 Department of Otolaryngology, New York Medical College, Valhalla, NY USA; 4 Department of Biological Engineering, Utah State University, Logan, UT USA; 5 Department of Dermatology, New York Medical College, Valhalla, NY USA

**Keywords:** Natural compounds, IgE, Allergy, Th2 response, Traditional Chinese medicine

## Abstract

IgE-mediated allergic diseases are common and greatly affect quality of life. Current therapeutic strategies include avoidance, which is unreliable, and unspecific symptom management, which causes adverse effects and are expensive. This urges the need for safe, effective, and affordable treatment options for infants, children, adolescents, and adults. This narrative review discusses naturally occurring compounds that have been researched in animal models, in vitro cell lines, and clinical trials to inhibit pathological IgE. These include Food Allergy Herbal Formula (FAHF)-1 and -2 and the derivatives B-FAHF-2, EBF-2, and berberine; *Rubia cordifolia* and its isolate xanthopurpurin; arctigenin; Anti-Asthma Herbal Medicine Intervention and its isolate formononetin; apigenin; luteolin; resveratrol; pterostilbene; and ginsenoside Rh1. Natural compounds largely work through reduction of IgE production of B cells through modulation of genes involved in B cell activation, differentiation and proliferation, and cell cycle. There is a resulting reduction of Th2 responses and mast cell degranulation, and hence allergic disease pathology and symptomology. There are rich resources of natural compounds, including those with the capacity to restore immunity to that seen in non-allergic individuals. Natural compounds are promising, novel treatment strategies for the vast sufferers of food allergy, allergic asthma, allergic rhinitis, and allergic eczema.

**Graphical Abstract:**

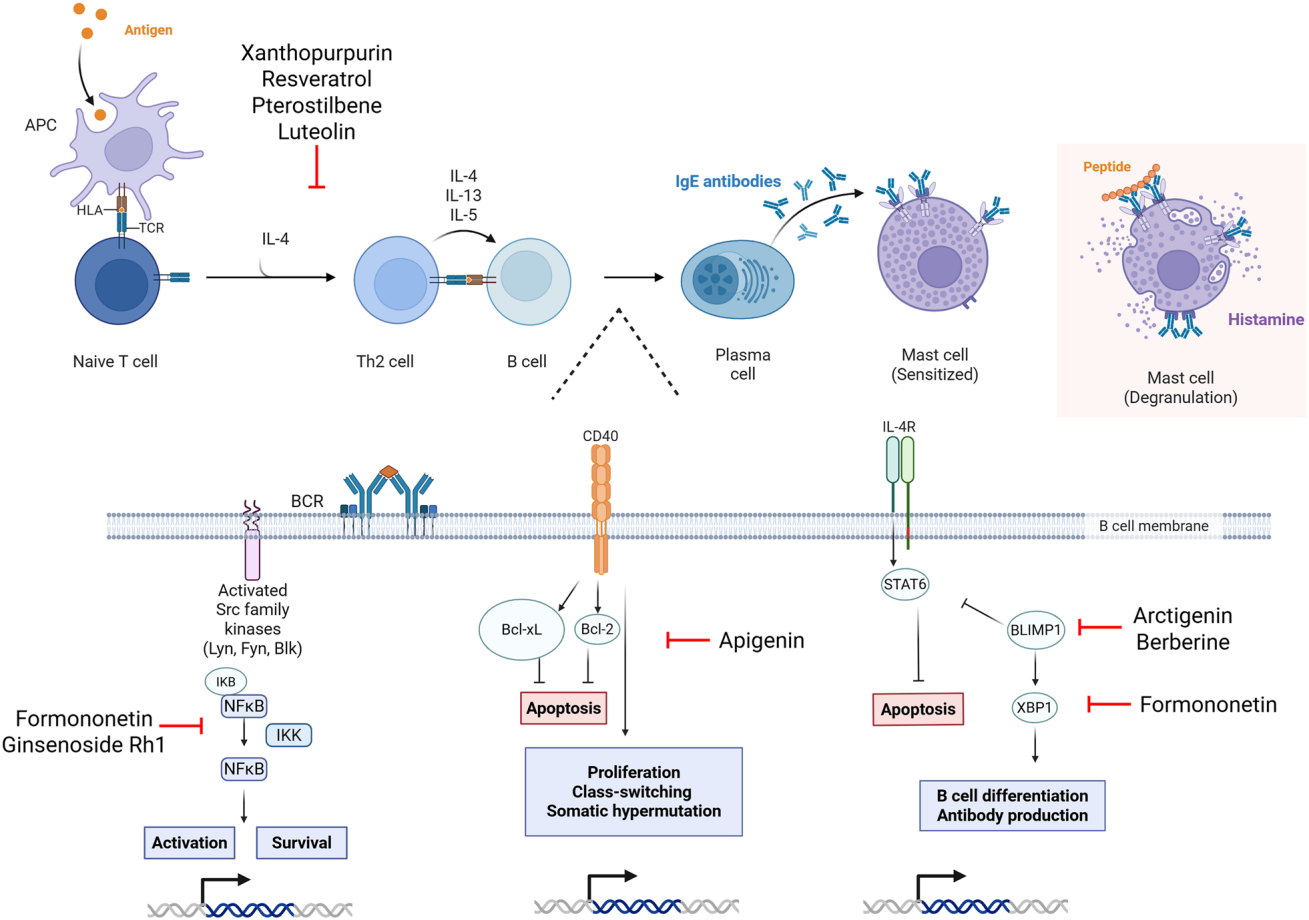

## Introduction

Immunoglobulin E (IgE)-mediated allergic diseases occur when the immune system mounts a response to an otherwise harmless antigen. The most common allergic diseases for all age groups include food allergies, allergic rhinitis, eczema (atopic dermatitis), and allergic asthma. The immune response mounted to allergens causes a variety of symptoms that occur because of the rapid release of inflammatory mediators, especially histamine, depending on how much is released and where [1]. Food allergies can trigger a range of symptoms, from minor skin irritation to life-threatening anaphylaxis. Allergic rhinitis causes sneezing and congestion. Eczema causes itching and rashes on the skin. Allergic asthma causes coughing, wheezing, and shortness of breath. While allergy awareness and education has improved, emergency department visits for anaphylaxis [2] and fatality from severe allergic reactions has not, and therefore the identification of treatments and interventions is a dire unmet need. Current strategies include trigger avoidance, medications including corticosteroids, and immunotherapy [1]. However, avoidance is unreliable and exacerbates fears of accidental exposure, resulting in severely diminished quality of life. Generalized management of symptoms after exposure includes antihistamines, antileukotrienes, steroids, and epinephrine, all of which have strong limitations across all allergic diseases [1].

Food allergen-directed immunotherapies function by inducing food desensitization, such as Palforzia for peanut allergy, with disadvantages including adverse reactions, ineffectiveness in adults and for severe cases, and relapses upon cessation of therapy. Omalizumab (Xolair), an anti-IgE biological, has been approved by the Food and Drug Administration (FDA), however, it blocks IgE without targeting its production, and hence sustained unresponsiveness does occur, like oral immunotherapy (OIT) [3, 4]. Epicutaneous immunotherapy is expensive [5], working by administering a small dose of peanut allergen to desensitize the patient. Immunotherapy needs standardization of protocols, demonstrated by their various administration routes, doses, and timing [1]. Therapies that induce long-term food tolerance are desired but have yet to be established.

Asthma treatment includes controllers, medications taken daily to prevent disease exacerbation, and relievers, those that are used as needed for immediate relief. Drawbacks include dose-dependent adverse effects and the development of steroid resistance. A subset of patients fails to adequately response to corticosteroids, inhaled or oral, which can contribute to severe disease progression [6, 7]. Biologicals are expensive and not readily available to all patients, and some patients do not even respond to them at all. Safe and effective treatment options are needed for those with allergic asthma.

Allergic rhinitis medications include antihistamines, which cause drowsiness and have evidence of cardiac toxicity, intranasal corticosteroid sprays, which increase the risk of nasal dryness, nosebleeds, rhinitis medicamentosa, anosmia, headache, and nasal septum perforation, and decongestants, which have rebounding effects after a few days [8–10]. Systemic corticosteroids should be used as a last choice, and only for the short term [11]. To combat these, repeated immunotherapy containing high doses of specific allergens can be used, with options including subcutaneous immunotherapy, also known as allergy shots, and FDA-approved sublingual immunotherapy tablets [12]. However, clinical benefits are conferred after at least three years [13–15]. Hence, safe and effective treatments for allergic rhinitis sufferers are crucial.

Medications for atopic dermatitis include both topical and systemic treatments. Topical treatments include moisturizers, corticosteroids, and calcineurin inhibitors, which are more effective for mild cases [16, 17]. Topical corticosteroids are efficient for maintenance therapy, however topical corticosteroid risks “addiction” or withdrawal syndrome (TSA/TSW). TSA/TSW increases the difficulty in symptom management, including erythroderma, sleep disturbance, and recurrent *Staphylococcus* infections [18]. Non-steroidal topical tapinarof (Vtama) is FDA-approved for patients aged two years and older, with side effects including upper and lower respiratory tract infections, folliculitis, asthma, headache, ear infection, vomiting, and abdominal pain [19]. Systemic options like dupilumab [20] and tralokinumab are approved by FDA for moderate-to-severe cases, however age limitations occur [21]. Phototherapy is also used, however long-term harmful effects include premature skin aging, hyperpigmentation, and increased risk for skin cancer [22]. This emphasizes the need for safe and effective treatment options for eczema patients.

The limited success of targeted medication can be attributed to multiple factors, including cytokine interactions of this complex and convoluted system with immunological redundancy, translatability of animal models to humans, individual responses to therapy, and side effects of these therapeutic agents [1]. The recurring message is that current therapeutic approaches urge the need for the identification of novel therapies that are lifesaving, long-lasting. and potentially curative. Traditional Chinese Medicine (TCM) is a promising therapeutic approach due to its demonstrated efficacy, affordability, and reduced risk of adverse effects [1]. Naturally occurring compounds exhibit immunomodulatory activity and may be harnessed for allergic diseases, serving as both preventative and treatment approaches. This narrative review is the first to focus on natural compounds that have demonstrated IgE suppression and regulation across common allergic diseases. Clinical trials, case studies, and primary articles have been investigated here for their use in IgE-mediated allergic diseases. These include Food Allergy Herbal Formula (FAHF)-1 and -2 and the derivatives B-FAHF-2, EBF-2, and berberine; *Rubia cordifolia* and its isolate xanthopurpurin; *A. lappa* and its minor compound arctigenin; Anti-ashtma Herbal Medicine Intervention (ASHMI) and its isolate formononetin; apigenin; luteolin; resveratrol; ginsenoside Rh1; and pterostilbene. There are rich resources of natural compounds, and excitingly, including those with the capacity to reduce IgE and restore immunity to that seen in non-allergic individuals. These natural compounds are promising and novel treatment strategies for the vast number of patients suffering from food allergy, allergic asthma, allergic rhinitis, and allergic eczema.

## Mechanism of allergic disease

**Fig. 1:**
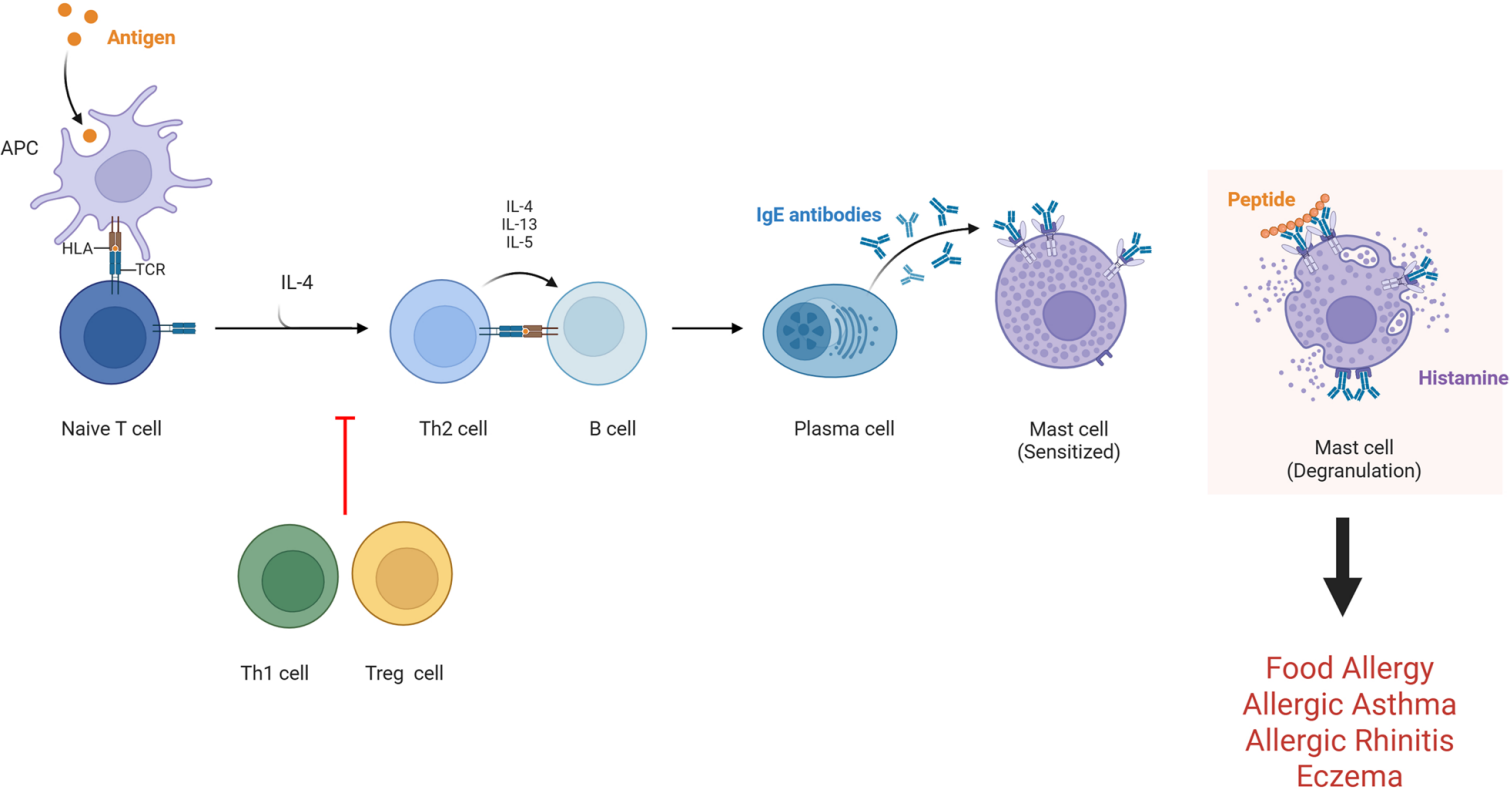
Cellular and molecular regulators of allergic diseases with the potential to be targeted by natural compounds. APC = antigen presenting cell; IgE = immunoglobulin E; IL = interleukin; Th = T helper, Treg = regulatory T cell. Adapted from Reference: [26] Complementary and Alternative Medicine for Treatment of Food Allergy by Xiu-Min Li, M.D.

IgE-mediated allergic responses are initiated by priming of T cells and dendritic cells toward Th2 phenotypes [1]. Th2 polarization yields B cell activation and their differentiation into IgE-producing plasma cells. Among Th2 cytokines, interleukin (IL)-4 drives B cell proliferation, while IL-5 facilitates B cell differentiation and the recruitment and activation of eosinophils within infected tissues [23]. IL-13 enhances epithelial cell turnover and mucus production at the site of inflammation [1, 23]. In addition, cytokines IL-3 and IL-9 attract mast cells to the infected tissue [24]. The secreted IgE can then bind FcεR1 on innate immune cells, such as mast cells, resulting in their sensitization. Upon re-encountering the antigen, IgE crosslinking initiates degranulation, releasing mediators such as histamine, which drive the acute manifestations of food allergy. While mast cells initiate the Th2 immune response, eosinophils dominate the anti-parasitic defense and chronic allergic reactions. Cooperating with mast cells through their own toxin and cytokine release, eosinophils amplify inflammation and constitute the late-phase response [25]. The release of major basic protein and cytokines IL-5, IL-3, and GM-CSF (granulocyte–macrophage colony-stimulating factor) from both mast cells and eosinophils activate basophils. Basophils, which constitutively express FcεRI, share effector molecules with mast cells and eosinophils. Collectively, these three cell types orchestrate both the early and late phases of IgE-mediated immunity (Fig. 1) [26]. Identifying novel TCM compounds that target these cells, molecules, and downstream signaling pathways has great impacts on allergy treatment and prevention.

## FAHF-2, B-FAHF-2, and EBF-2 for IgE-mediated allergic diseases

FAHF-1 is a traditional Chinese formula composed of eleven herbs including *ling zhi* (*Ganoderma*), *fu zi* (*Aconiti lateralis radix praeparata*), *wu mei* (*Mume fructus*), *chuan jiao* (*Zanthoxylum pericaprium*), *xi xin* (*Asari radix et rhizoma*), *huang lian* (*Coptis rhizoma*), *huang bai* (*Phellodendri cortex*), *gan jiang* (*Zingiberis rhizoma*), *gui zhi* (*Cinnamomum ramulus*), *ren shen* (*Panax ginseng*) and *dang gui* (*Angelica sinesis radix*). Due to potential toxicity upon improper processing, *xi xin* and *zhi fu zi* were later removed for formulation of the nine-herbal improved FAHF-2. FAHF-2 consists of 28.17% *ling zhi*, 28.17% of *wu mei*, 1.41% *chuan jiao*, 8.46% *huang lian*, 5.63% *huang bai*, 8.45% *gan jiang*, 2.81% *gui zhi*, 8.45% of *ren shen,* and 8.45% of *dang gui* [27]. The gastrointestinal actions of FAHF led to their assessment as a food allergy treatment strategy. High-performance liquid chromatographic (HPLC) fingerprints of FAHF-2, B-FAHF-2, and individual herb extracts were generated for product quality control and standardization according to guidelines issued by the US Food and Drug Administration Guidance for Industry Botanical Drug Products [28–31].

### Food allergy

FAHF-1 containing *ling zhi*, an herb with anti-inflammatory and anti-allergy properties, and *wu mei wan*, used to treat colic, vomiting, and chronic diarrhea, was assessed as a food allergy remedy in vivo. Intragastric gavage of FAHF-1 (21 mg/mouse) inhibited peanut-induced anaphylaxis and peanut specific (s)-IgE by 2 weeks of treatment that persisted over 4 weeks post-therapy [32]. Lymphocyte proliferation and levels of IL-4, IL-5, and IL-13 were also reduced [32]. An improved 9-herbal formula, FAHF-2, whereby *zhi fu zi* and *xi xin* were eliminated, was tested for efficacy compared to FAHF-1 [33]. Intragastric FAHF-2- treatment (20 mg/mouse) prevented anaphylaxis, plasma histamine elevation, and vascular leakage in peanut allergic (PNA) mice [33]. The levels of IgE significantly declined by FAHF-2 treatment and persisted at 5 weeks post-therapy [33]. Peanut stimulation of the splenocytes of FAHF-2-treated mice produced less IL-4, IL-5, IL-13, and more interferon (IFN)-γ levels [33] demonstrating the effective repression of Th2 responses that may also be safe for peanut allergies. The prevention of anaphylaxis by FAHF-2 in PNA mice and whether protection was sustained after discontinuation of therapy, was conducted [34]. FAHF-2 treatment prevented anaphylaxis, reduced sIgE levels, and augmented IgG2a levels [34]. Cell of the FAHF-2 treated mice mesenteric lymph nodes (MLNs) secreted less IL-4 and IL-5 and more IFN-γ, which coincided with more IFN-γ-producing CD8 + T cells [34] confirming a long-term shift of Th2 and Th1 milieu in response to FAHF-2 treatment in vivo.

**Fig. 2:**
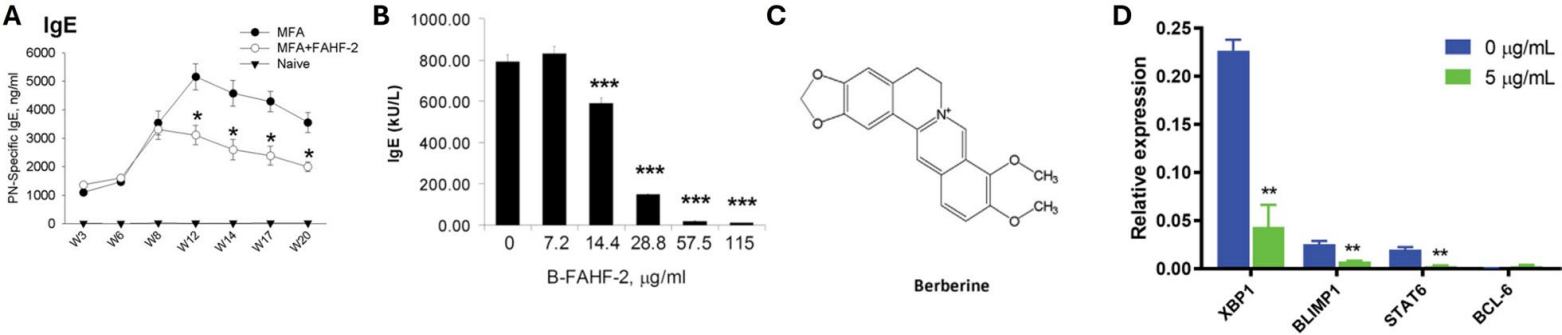
The effect of FAHF-2, B-FAHF-2, and BBR on IgE-mediated food allergy. The effect of FAHF-2, B-FAHF-2 and BBR on IgE-mediated Food Allergy. **A** FAHF-2 treated reduced peanut (PN)-specific IgE in in vivo models of food allergy [36]. **B** B-FAHF-2 reduced secretion of IgE in myeloma cells, U266, in vitro [30]. **C** Berberine structure. **D** RNA Seq analysis of PN allergic PBMCs demonstrated reduced IgE levels mediated by genes involved in B cell activation, differentiation and proliferation, including XBP1, BLIMP1, STAT6, and BCL-6 [48, 49]

To elucidate the long-term feasibility and mechanism of its persistence, FAHF-2 mediated protection was assessed [35]. FAHF-2 treatment (64 mg/mouse) in PNA murine models were protected from anaphylaxis, which lasted over 36 weeks post-treatment, accompanied by reduced peanut sIgE and increased IgG2a [35]. Th2 cytokine production by CD4 + T cells decreased, while IFN-γ production by CD8 + T cells increased in FAHF-2-treated mice from allergen-specific immune responses to beneficial responses by FAHF-2 [35]. With the success of FAHF-2 against anaphylaxis in murine PNA models, research into the effect on multiple food allergies (MFA) was done. MFA mice treated with FAHF-2 did not experience anaphylaxis or change in body temperature following challenges with sensitized allergens [36]. Immunologically, allergen sIgE levels, IL-4, and IL-13 levels were decreased and IFN-γ levels were increased in FAHF-2 -treated mice (Fig. 2A) [36]. FAHF-2 has great potential for treating human MFA.

The success of the nine-herbal formula of FAHF-2 warranted investigation into the pharmacological and immunological effects of individual herbs to determine if all nine are necessary, or if a simpler formula is equally effective. It was evident that some individual herbs were able to decrease anaphylaxis in murine models, however no single herb reached the same level as FAHF-2 [37]. Individual herbs had varied effects on histamine release, peanut sIgE, and IgG2a levels [37]. A simplified formula of the most effective ones only showed partial efficacy, again not comparable to the protection of FAHF-2, and implying that all nine herbs may function synergistically to drive the therapeutic benefits observed in vitro and in vivo [37].

A randomized, double-blind, placebo-controlled, dose escalation phase 1 clinical trial was conducted to assess the safety and tolerability of FAHF-2 in food allergy patients (2.2 g, 3.3 g, or 6.6 g, 3 times a day for 7 days). In the FAHF-2- treated patients, IL-4 levels were significantly reduced [29]. In vitro studies of FAHF-2-treated peripheral blood mononuclear cells (PBMCs) (100 or 250 μg/mL) also showed significant declines in IL-5 levels and rises in IFN-γ and IL-10 levels [29]. FAHF-2 exhibited a favorable safety and tolerability profile in patients with food allergy. An extended phase 1 study investigating the immunological effects of FAHF-2 on peripheral blood basophils in food allergy patients was conducted. FAHF-2 (3.3 g) was well tolerated, safe, and inhibited basophil CD63 expression in response to ex vivo stimulation at 6 months, warranting a controlled phase 2 study [28]. The safety and efficacy of FAHF-2 for food allergy treatment was assessed in a double-blind, randomized, placebo controlled study (5 g, three times a day for 6 months). Clinically, the FAHF-2 was well-tolerated and no severe adverse reactions occurred, however epinephrine required for reaction treatment was the same between both groups [38]. Immunologically, there were no significant differences in sIgE, IgG4, PBMC cytokine production, or basophil activation between groups [38]. However, in vitro treatment of baseline PBMCs from subjects that were treated with FAHF-2 produced significantly less IL-5 and more IL-10 and Tregs when compared to untreated [38]. Immunological insignificance may have been due to low drug adherence for over one-third of the study duration.

As the FAHF-2 pill-load is not favorable for pediatric clinical studies, the butanol-purified extract of FAHF-2 (B-FAHF-2) was tested for efficacy, safety and immunological mechanisms in vivo. By butanol purification, the effective dose reduced about fivefold (12 mg/mouse per day) [30]. B-FAHF-2 demonstrated protection from anaphylaxis in a PNA mouse model, with partial protection persisting for up to 50 weeks [30]. Immunologically, Th2 cytokines, IgE, and histamine levels were reduced in vivo [30]. In vitro, PNA splenocytes and MLNs had reduced IL-5, IL-4, IL-13, and IL-10 production while IFN-γ was significantly increased upon B-FAHF-2 treatment [30]. Similar, B-FAHF-2- treated peanut-polarized splenocytes had inhibited the production of IL-4 and IL-5 without causing cell cytotoxicity [30]. The reduction in IgE levels was confirmed in myeloma B cells, U266 (Fig. 2B) [30]. The improvement of peanut/tree nut OIT adverse reactions and enhanced persistence of food tolerance by B-FAHF-2 (BF2) was investigated in vivo. Mice treated with both OIT and BF2 (12 mg/mouse per day) demonstrated significantly less severe and fewer adverse reactions than OIT-treated, alone [39]. While both groups showed significant desensitization alone, the mice treated with both OIT and BF2 were desensitized to a higher extent [39]. In agreement with the clinical outcomes, markedly lower plasma histamine and IgE levels, augmented IFN-γ/IL-4 and IL-10/IL-4 ratios, re-methylation of the IL-4 promoter and de-methylation of the IFN-γ and Foxp3 promoters were observed [39]. BF2 in combination with OIT was safer with better clinical and immunological outcomes than OIT, alone, in vivo.

Mast cells are a key player in IgE-mediated allergic disease, therefore with the protection induced against peanut anaphylaxis in mice by FAHF-2, the mechanism underlying this effect was sought out by Song et al. by investigation of mast cell/basophil numbers and IgE-mediated activation in PNA mice. After 7 days of treatment (64 mg/mouse per day), peripheral blood basophil number reduced, and maintained for at least 4 weeks after therapy [40]. By 4 weeks post-therapy, the number and FcεR1 expression of peritoneal mast cells significantly declined, and this was confirmed in vitro in FAHF-2—treated MC/9 cells [40]. MC/9 cells treated with FAHF-2 also demonstrated significantly reduced FcεR1γ mRNA subunit expression, proliferation, and histamine release upon challenge [40]. Similarly, in human mast cells FAHF-2 (20 μg/mL) treatment inhibited degranulation [40]. To identify active compounds, FAHF-2 was extracted and divide into 4 fractions, with a demonstrated superiority in fraction 2 against rat basophilic leukemia (RBL-2H3) degranulation (18 μg/mL and 36 μg/mL) [40]. Three compounds from this fraction- berberine (BBR), palmatine, and jatorrhizine- suppressed tyrosine kinase phosphorylation attributed to the reduction in degranulation [40]. Coincidingly, the noncytotoxic reduction in RBL-2H3 degranulation by B-FAHF-2 (2 μg/mL) was established [30].

FAHF-2 and B-FAHF-2 were also examined for their effects on pediatric Crohn’s disease. FAHF-2-treated mice had improved clinical and immunological parameters, including decline in weight loss, improvement in histology, and inhibition of tumor necrosis factor (TNF)-α, IL-17, IL-6, and IFN-γ production [41]. Similarly, in vitro treated PBMCs produced less TNF-α, IFN-γ, and IL-12, with evidence of decreased TNF-α producing monocytes and T cells [41]. When B-FAHF-2 was compared to FAHF-2 and the individual herbs, similar to what is seen in food allergy, B-FAHF-2 was more efficacious, at 1/5 of the dose, than FAHF-2 at reducing TNF-α production and increasing GM-CSF production by PBMCs and colonic mucosa from pediatric Chron’s disease subjects [31]. Only one of the nine individual herbs demonstrated similar immunological effects [31]. In vivo, treatment with B-FAHF-2 alleviated colitis [31]. Consistent with the results seen in in vitro and animal model studies, clinical study of FAHF-2 and its derivative showed high safety profiles consistently observed [28, 29, 42] and beneficial immunological responses such as the suppression of Th2 cytokines (IL-5), reduced milk and peanut allergen skin test reactions as compared to placebo controls, and reduced basophil activation, as compared with baseline [42]. However, clinical protection has yet to be determined [28]. Improvement of retention rates by increasing the convenience and optimizing the dosing, including improved oral bioavailability, may lead to stronger clinical outcomes [43].

### Pharmacokinetics and bioavailability

Pharmacokinetic and bioavailability data for the complete FAHF-1, FAHF-2, B-FAHF-2, and EBF-2 formulations are limited, and hence the characterization of absorption, distribution, metabolism, and elimination have yet to be established. While B-FAHF-2 retained safety and efficacy at 1/5 the FAHF-2 dose, the isolation of and consequential investigation into BBR was performed for its role as a chemical and pharmacokinetic marker [44]. This is common for multi-herbal formulations whereby pharmacokinetics is greatly impacted by synergy, altered metabolism, or tissue targeting. When mice were treated with B-FAHF-2 or BBR at the equivalent concentration, anaphylactic protection was lost and pharmacokinetics showed the maximal plasma concentration (Cmax) of B-FAHF-2 was 289.30 ± 185.40 ng/mL and BBR, alone, showed very low bioavailability with Cmax value of 35.13 ± 47.90 ng/mL [44]. This was confirmed in vitro, with Caco-2 cells influx demonstrating the combination of herbal constituents increases absorption [44]. Authors confirmed that BBR can serve as a chemical and pharmacokinetic marker of B-FAHF-2 [44]. Further studies evaluating the pharmacokinetics of complete formulations are warranted.

## Berberine for IgE-mediated allergic diseases

Berberine is an organic isoquinoline alkaloid found in many medicinal plants. It has a molecular weight of 336.4 g/mol and a molecular formula of C_20_H_18_NO_4_^+^ (Fig. 2C). BBR is a yellow crystalline powder obtained from medicinal plants. BBR has demonstrated diverse pharmacological effects including antilipemic, antioxidant, and antineoplastic, to name a few. With that, BBR has been used orally as an anti-fungal and antiparasitic, and as an antidiarrheal. In various studies across different diseases and conditions, it has been demonstrated that BBR is clinically safe and well-tolerated in humans, with mild gastrointestinal side effects [45–47].

### Food allergy

An additional study set out to determine the constituents of FAHF-2 and B-FAHF-2 that specifically reduce IgE. Both compounds decreased the production of IgE by IgE-producing plasma cells, U266 with B-FAHF-2 9 × more effective than FAHF-2 (78% at 500 μg/mL vs 92% at 120 μg/mL) [48]. Isolated from *Philodendron chinesis*, BBR and limonin inhibited PBMC IgE production, with BBR being more potent (80% at 20 μg/mL) [48]. BBR inhibited their IgE germline transcript expression, phosphorylated IκBα, and increased expression of TBX21 (T-bet) and signal transducer and activator of transcription (STAT)-3 [48]. As B-FAHF-2 (12 mg/mouse per day) retained the safety and efficacy of FAHF-2, isolation and identification of the major bioactive component present in both confirmed BBR. As described above, in vivo, BBR (1 mg/mouse per day) alone demonstrated low bioavailability compared to B-FAHF-2, therefore other components of B-FAHF-2 may facilitate BBR uptake (18–205%) [44].

A purified version of FAHF-2, called EBF-2, was developed by Yang et al. EBF-2 inhibited IgE production 16× more than FAHF-2 in vitro and these effects were evident in a dose-dependent manner (IC_50_ = 4.70 μg/mL) [49]. In PNA mice, EBF-2 treatment (40 mg/mL for 14 days) decreased peanut sIgE and the amount of IgE-producing plasma cells, and presented clinically with complete protection from anaphylaxis and plasma histamine secretion upon peanut challenge [49]. No effects on IgG1 or IgG2a production were observed [49]. BBR, the active component, was calculated to be 0.36% of FAHF-2 and 4.4% of EBF-2. BBR dose-dependently inhibited IgE reduction in a non-cytotoxic manner (nearly 100% at 5 μg/mL) and at 5 μg/mL, inhibited X box binding protein 1 (XBP1), B-lymphocyte-induced maturation protein 1 (BLIMP1), and STAT6 suppression, and reduced mitochondrial oxidation rate of IgE-producing plasma cells in vitro (Fig. 2D) [49]. This was confirmed in human tonsil samples from patients, with BBR treatment (10 μg/mL) significantly decreasing IgE production upon stimulation in a dose-dependent manner, without affecting IgG production or cell viability [50]. The results suggested that BBR modulates IgE expression at the transcriptional level, through inhibition of STAT6 binding through B cell lymphoma (BCL)-6 at the IgE heavy chain promoter [50]. This group further elucidated this IgE-specific mechanism by BBR at 10 μg/mL, highlighting the miRNA (miR) 34a- p53 axis imperative to B cell development and pathological IgE production [51].

The success of BBR on mitigation of the production of IgE and the subsequent Th2 response seen in allergy was extended into the effects on the gut microbiota. PNA mice receiving BBR-containing (2 mg/ mouse per day) oral boiled peanut immunotherapy (BNP) demonstrated enduring tolerance to peanut with sustained reductions in IgE, plasma histamine, symptom scores including body temperature, and amount of IgE + B cells [52]. As gut microflora influence food allergy, there were also observed differences in bacteria genera, including positive correlation with *Lachnospiraceae*, *Ruminococcaceae*, and *Hydrogenanaerobacterium* (all *Firmicutes*), and negative correlation with *Verrcromicrobiacea, Caproiciproducens, Enterobateriaceae,* and *Bacteroidales* [52]. BNP is promising as a food allergy treatment regimen and the associated distinct microbiota signature in vivo.

### Allergic asthma and allergic rhinitis

An in vitro model of allergic airway inflammation, BEAS-2B human bronchial epithelial cells, were pre-treated with BBR (1 μM) and activated, resulting in significant inhibition of IL-6 and C–C motif chemokine ligand (CCL)-11 secretion, possibly through suppression of STAT6 signaling, without affecting cell viability [53]. To assess the effect of BBR on allergic inflammation in allergic rhinitis in vivo and examine its mechanism of action, murine models of *Dermatophagoides farinae* (Derf)-sensitized allergic rhinitis were administered BBR (Derf + BBR) or untreated (Derf, only) at 10 μg/mL [54]. In BBR-treated groups, symptom scores, sIgE, GATA binding protein -3 (GATA-3) mRNA, T-bet mRNA, decreased while the percentage of CD4 + CD25 + Foxp3 + T cells increased [54]. In mice co-administered anti-CD25 mAb and BBR, serum IL-10 and Foxp3 mRNA levels were also decreased [54]. This group also saw reduced symptom scores, serum IgE, eosinophil infiltration and increased CD4 + CD25 + Foxp3 + T cells [54], suggesting BBR reduces allergic inflammation potentially in a T-cell dependent mechanism, as evident but their alteration in number in function. In another in vivo allergic rhinitis model, ovalbumin (OVA)-induced rats that were administered BBR (100 mg/kg) and CoQ10 demonstrated reduced nasal symptom scores, plasma IgE, IL-4, malondialdehyde, and nitric oxide (NO) levels and coincided with significant improvement in inflammation in nasal tissues subjected to histopathological immunohistochemical staining [55].

Specific molecular mechanisms and therapeutic targets were not yet clear, thus Luo et al. integrated bioinformatic analysis with in vivo experiments for validation utilizing OVA-induced guinea pigs. In silico analysis from publicly available murine model datasets that may be associated with BBR demonstrated pathways including Nuclear Factor kappa B (NF-κB), IL-17, TNF, and inflammatory response [56]. Strong affinity via molecular docking analysis of key genes Alb, IL-6, IL-1β, toll-like receptor (TLR)-4, Prostaglandin-Endoperoxide Synthase 2 (PTGS2; encodes cyclooxygenase-2 (COX-2)) was elucidated [56]. In guinea pig models in vivo, confirmed in silico analysis, as mitigation of allergic rhinitis symptoms including sneezing and rubbing, and reductions in serum levels of IL-17, IL-6, TNF-α, and IL-1β were observed upon BBR treatment (50 and 100 mg/kg) [56]. Downregulation of IL-6, TLR4, PTGS2, and IL-1β expression were also a consequence of BBR treatment [56]. The anti-inflammatory effects of BBR were extended into the airway inflammation of asthma. In vivo rat models of OVA-induced asthma demonstrated significant dose-dependent (100 mg/kg and 200 mg/kg) reductions in inflammatory cells in bronchoalveolar lavage fluid (BALF), lung inflammation scores, and NF-κB signaling activating promoted by IgE production [57].

### Atopic and contact dermatitis

Atopic dermatitis (AD) is a chronic inflammatory disease of the skin, therefore Andoh et al. investigated the anti-inflammatory effects of BBR atopic dermatitis in vivo. BBR treatment (50 mg/kg, 100 mg/kg) of NC/Nga mice prevented skin reactions including itching and the infiltration of eosinophils and mast cells, characterized by the reduced expression of cutaneous eotaxin, macrophage migration inhibitory factor and IL-4 [58]. An increase in eukaryotic translation initiation factor 3 subunit F (EIF3F) and Mucosa-Associated Lymphoid Tissue Lymphoma Translocation 1 (MALT1) was also demonstrated to be reduced by BBR treatment of murine mast cells (100 μM), suggesting that through inhibition of eotaxin and pro-inflammatory cytokines and the inflammatory cells recruited, BBR is able to improve AD symptoms, and this may be due to the downregulation of EIF3F and MALT1 [58].

Eczema sufferers are prone high incidence of *Staphylococcus aureus* (*S. aureus*) skin colonization, which stimulates macrophages and the subsequent release of pro-inflammatory cytokines and mediators, therefore effects of BBR treatment were investigated. Murine macrophage RAW264.7 and human monocyte U937 cells were treated with BBR and heat killed *S. aureus* (HKSA) or *S. aureus* derived from severe eczema patients undergoing topical steroid withdrawal. BBR demonstrated bacteriostatic effects on both *S. aureus* from American Type Culture Collection (5–20 μg/mL) and clinical isolates (64–512 μg/mL) and suppressed TNF-α production in both in vitro cell lines exposed to HKSA in a dose-dependent noncytotoxic manner [59]. In U937 cells, BBR (20 μg/mL) downregulated genes of inflammatory pathways including advanced-glycation end products (AGE)- receptors for AGEs (RAGE) and IL-6, PTGS2, caspase (CASP)3, mitogen-activated protein kinase (MAPK)-1, and IL-1β, and inhibited *S. aureus* reactive oxygen species (ROS) production in vitro [59].

### Pharmacokinetics and bioavailability

As described above, BBR has a low bioavailability and poor oral absorption, at less than 1% across various in vivo and in vitro studies [60]. This was confirmed in humans and thought to be attributed to the extensive intestinal first-pass elimination increasing its metabolism [60]. To address the low water solubility and bioavailability, a platelet membrane (PM)-coated nanoparticle (NP) system was developed for targeted delivery of BBR (PM@Ber-NP) to inflammatory lungs of an house dust mite (HDM)-induced asthma mouse model [61]. Nasal delivery of PM@Ber-NP (2 mg/kg) prevented lung inflammation compared to free BBR, evident by the reduced inflammatory cells and cytokines in the BALF sera and lungs [61]. The reduction in IL-4, IL-5, and IL-13 coincided with enhanced IL-12 expression, indicating regulation of the Th1/Th2 balance [61]. Notably, the loading of BBR into nanoparticle carriers could delay the release of BBR compared with free BBR and facilitate steady drug release for about 24 h, which could overcome bioavailability barriers in humans. BBR appears to be rapidly distributed, with the potential for novel technology to further establish its distribution and other pharmacokinetic profile, in addition to its nanotechnology carriers [60].

## *Rubia cordifolia* and xanthopurpurin for IgE-mediated allergic diseases

*Rubia cordifolia* is a perennial botanical drug climbing vine, with the rhizome demonstrating high clinical significance. Phytochemical analysis has reported over 100 compounds including terpenes, flavonoids, and quinones, and multiple pharmacological activities including neuroprotective, anti-neoplastic, antibacterial, anti-inflammatory, antioxidant, and immunosuppressive [62].

### Food allergy

**Fig. 3:**
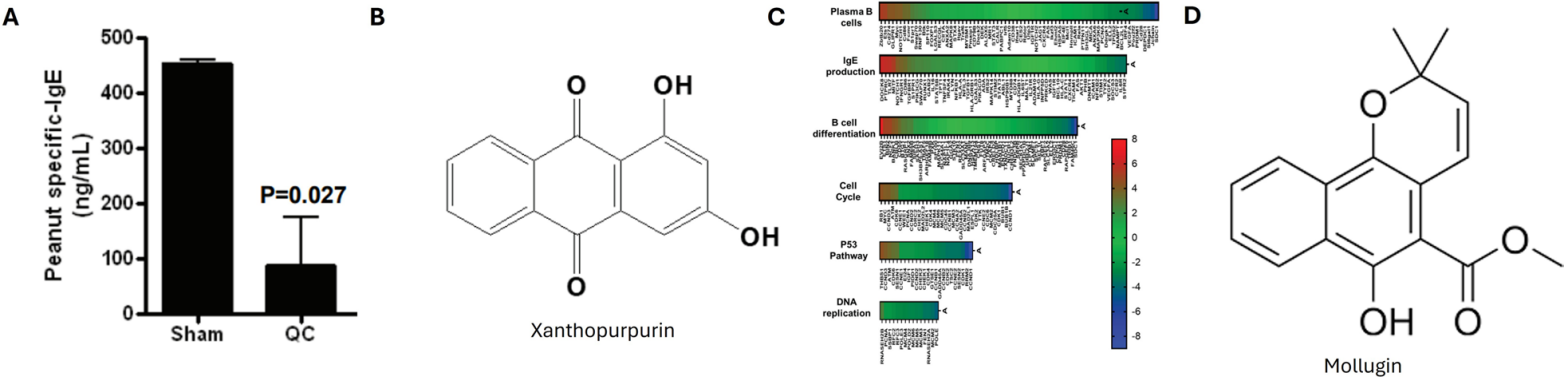
The effect of Rubia cordifolia and its isolate, xanthopurpurin (XPP), on IgE-mediated food allergy. **A** *R. cordifolia* (QC) inhibited PN sIgE levels in vivo [63]. **B** XPP structure. **C** XPP –treated U266 cells demonstrated altered transcriptomics highlighting genes involved in plasma B cells, IgE production, B cell differentiation, cell cycle, p53, and DNA replication, including CCND1, SDC1, IL6R, DUSP4, ETS1, and PTPRC [64]. **D** Mollugin structure

The testing of several herbal extracts for the ability to reduced IgE production in vitro (100 and 500 μg/mL), yielded the identification of *Dianthus superbus* and *Rubia cordifolia* [63]. In a dose-dependent manner (3.125–100 μg/mL), both compounds reduced IgE production in the U266 human B cell line [63]. In murine models of PNA, IgE production was reduced (Fig. 3A) without affecting IgG1 levels when treated with *R. cordifolia* (4 mg/mouse) [63]. Anaphylaxis and plasma histamine levels induced with peanut challenge were reduced, suggesting their potential for allergy treatment [63].

The water extract of *R. cordifolia* was assessed for compounds with bioactivity against IgE production. Xanthopurpurin (XPP) was identified and purified to determine its effects i*n vitro* and in vivo*.* Xanthopurpurin (1,3-dihydroxyanthraquinone) is an anthraquinone in *R. cordifolia*. XPP has a molecular weight of 240.2 g/mol and a molecular formula of C_14_H_8_O_4_ (Fig. 3B) that has demonstrated antiviral, antiplatelet, antioxidant, and immunomodulatory activities. Human IgE-producing myeloma U266 cells demonstrated significant and dose-dependent reductions in IgE production with XPP treatment (IC_50_ = 9 μg/mL) [64]. In a murine model of food allergy, XPP (400 μg/ mouse per day) significantly reduced peanut sIgE, plasma histamine levels, and allergic reactions [64]. This protective effect was evident upon cessation of XPP treatment [64]. XPP treatment significantly reduced IL-4 levels and increased IL-4 promoter methylation, without affecting IgG, IgA, or IFN-γ production [64]. XPP treatment also reduced peripheral and bone marrow IgE + B cells [64]. XPP treatment altered expression of genes involved in B cell differentiation and plasma cell IgE production, including cyclin d1 (CCND1), dual specificity phosphatase 4 (DUSP4), syndecan -1 (SDC1), erythroblastosis virus proto-oncogene 1 (ETS1), protein tyrosine phosphatase, receptor type, C (PTPRC), and IL-6R (Fig. 3C) [64]. XPP was deemed safe through assessment of mortality, morbidity, and blood biochemistry and CBC analysis at both a 10× dose (4 mg/mouse per day for 14 days) and 5× sub-chronic assessment (2 mg/mouse per day for 14 days) [64]. *Rubia cordifolia* compound XPP may be a potential therapy for IgE-mediated food allergy.

### Allergic asthma and allergic rhinitis

Manjishthadi Kwatham (brihat) (MMK) is a polyherbal formula of which *R. cordifolia* is a constituent, and treatment of mouse models of acute anaphylaxis and mast cell degranulation, guinea pig models of allergic rhinitis, goat red blood cells (RBC), and Chinese hamster ovary (CHO) cells were performed [65]. MMK treatment (0.25 g/kg per day for 14 days) reduced 1% evans blue dye leakage in both mouse models, sneezing and blood eosinophil count in guinea pigs, attenuated plasma histamine, protected RBCs, and inhibited CHO intracellular calcium release [65]. An isolate of *Rubia cordifolia*, mollugin, has demonstrated anti-inflammatory activity and was investigated for its effects on allergic airway inflammation. Mollugin (1-hydroxy-2-methy-9,10-anthraquinone; C_17_H_16_O_4_, 284.3 g/mol, Fig. 3D) is an anthraquinone, like XPP. Mollugin (5 mg/kg, 10 mg/kg per day) attenuated in vivo eosinophil infiltration, lung eosinophil peroxidase activity, and epithelial mucus secretion in lung tissues of ST-induced asthma murine models [66]. Mollugin also lowered secretion of IL-4 and IL-5, and mRNA levels of IL-4, IL-5, IL-13, eotaxin, CCL-17, Mucin (Muc) 5ac, arginase-1, Ym-1 and Fizz-1 in lung tissues in a non-cytotoxic manner [66]. In silico analysis proposed the mechanism of action of mollugin equates to that of a p38 MAPK inhibitor or poly(ADP-ribose) polymerase (PARP)-1 inhibitor [66]. Immunohistochemistry confirmed arginase-1 in the lungs and macrophages in the BALF and demonstrated arginase-1 mRNA expression and p38 MAPK phosphorylation were inhibited in peritoneal macrophages stimulated with IL-4 [66]. The mouse primary splenocytes treated with mollugin also had reduced IL-4 and IL-5 production with downregulated PARP1 and PAR protein levels [66]. Mollugin (15–30 μM) has also demonstrated apoptosis induction of human Jurkat T cells via c-Jun N-terminal Kinases (JNK) and CASP-12 [67]. *Rubia cordifolia* component mollugin may ameliorate allergic airway inflammation by inhibition of the Th2 response and macrophage polarization.

### Pharmacokinetics and bioavailability

As with complete herbal formulas, the whole R. cordifolia herb as a whole has not been largely investigated for its pharmacokinetic profile and bioavailability, however studies have addressed its main constituents, including mollugin, purpurin, and XPP. After oral administration of 0.82 g/kg of R. cordifolia extract, the Cmax were 70.10 ± 11.78 ng/mL for purpurin and 52.10 ± 6.71 ng/mL for mollugin, and the time for maximal concentration (Tmax) was 1.61 ± 0.24 h for purpurin and 1.99 ± 0.21 h for mollugin [68]. To determine its metabolites and circulating forms, purpurin was also identified from plasma and urine in another study following oral administration of R. cordifolia extract [69]. The differences were thought to potentially be due to their concentrations within the extract. Treating with XPP at 10 mg/kg orally, the bioavailability was low, at 4.6% [70]. To date, XPP-specific formulation studies to improve bioavailability are limited, however platforms like nanoparticle delivery are used to improve poorly soluble anthraquinones [71, 72].

## *Arctium lappa* and arctigenin for IgE-mediated allergic disease

*Arctium lappa* fruit has been used in traditional medicine, exhibiting antioxidant, anti-inflammatory, and anti-cancer effects. This biennial medicinal plant belongs to the Asteraceae family and is found all over the world. The roots and seeds have demonstrated various pharmacological effects, with the mature seeds’ effects primarily due to arctigenin. Arctigenin is the aglycone of the major compound within the plant, arctiin. Arctigenin has a molecular formula of C_21_H_24_O_6_ and a molecular weight of 372.4 g/mol. It is a lignin with anti-neoplastic, anti-inflammatory, and anti-infectious properties. Oral administration of the *A. lappa* plant (50 mg/kg, 250 mg/kg) has been evaluated for safety and was deemed “very safe” with therapeutic effects including the suppression of body weight gain and blood glucose [73]. In a clinical trial of an Canvalia gladiata- A. lappa extract (600 mg each) complex, it was determined to be safe and efficacious, enhancing immune functions through the IL-10 mediated stimulation of NK cells [74]. Although the safety literature for arctigenin is diverse, the preponderance of well-conducted studies supports its safety, whereas studies reporting adverse effects are limited by methodological shortcomings [75–78]. The methodological limitations outlined by Li et al. could substantially impact the interpretation of the results, as their PEG400 vehicle control did not completely dissolve arctigenin, and their vehicle control demonstrated effects on the organ systems investigated [75] which is expected of PEG400 upon literature review [79, 80]. Their conclusion was criticized by Mei et al., stating “it was unscientific to discuss toxicity without specifying the dose or range. It is crucial to identify the optimal dose range that produces significant effects and the lowest observed adverse-effect level of arctigenin.” [77]. In another study, performed by the same group, their interpretation of their findings appears inconsistent with the data presented, with misleading claims [81]. The study should not conclude 12 mg/kg of arctigenin as the lowest observed adverse effect level because their high dose groups (36 mg/kg, 120 mg/kg) exhibited no toxicity. A high safety profile agrees with a Phase 1 clinical trial of GBS-01, an orally administered drug rich in arctigenin, whereby no dose-limiting toxicities were observed [82]. Further studies are needed to define the optimal dose range of arctigenin and generate a no observed adverse effect level and a lowest observed adverse effect level.

### IgE-mediated mast cell degranulation

**Fig. 4:**
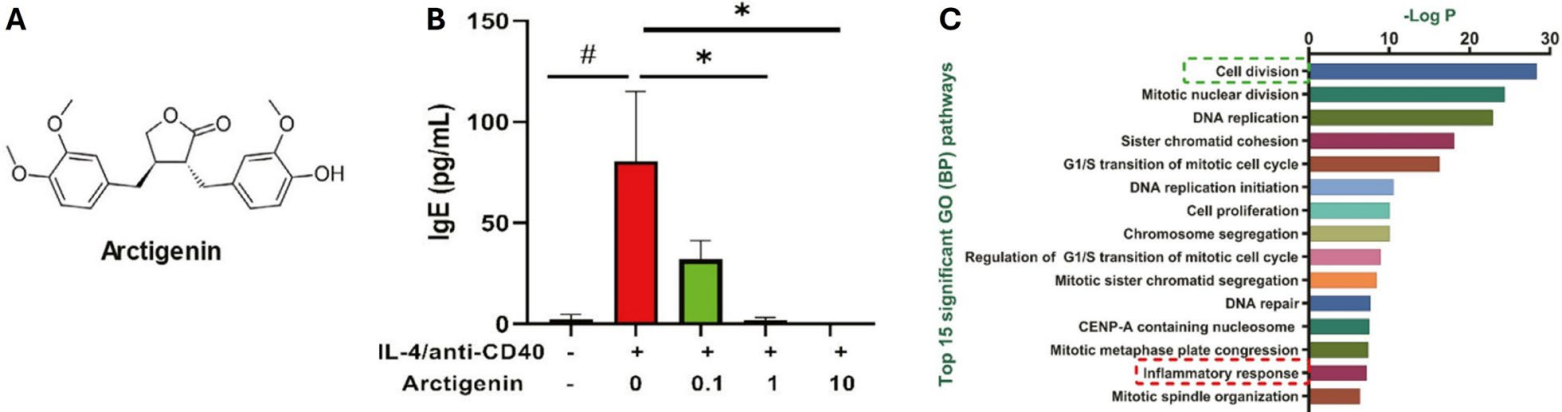
The effect of arctigenin on IgE-mediated food allergy. **A** Arctigenin structure. **B** Peanut allergic human PBMCs treated with arctigenin showed reduced IgE expression upon stimulation compared to untreated controls [76]. **C** RNA Seq of these treated- PBMCs highlighted alterations in cell division and cell cycle-related genes and inflammatory response related genes [76]

*Arctium lappa* fruit extract (AFE) and its fermented form (F-AFE) were studied for its effects on allergic responses, specifically mast cell degranulation. AFE is a complex mixture characterized by key active compounds including arctigenin, arctiin, chlorogenic acid, and caffeic acid. F-AFE significantly reduced β -hexosaminidase release (IC_50_ = 30.73 µg/mL), TNF-α (IC_50_ = 46.96 µg/mL), and PGE2 (IC_50_ = 36.27 µg/ml) production by IgE-activated RBL-2H3 mast cells in a noncytotoxic and dose-dependent manner [83]. F-AFE inhibited Lyn, Fyn, Syk phosphorylation, involved in FcεR1 signaling, and phosphoinositide phospholipase C (PLC) γ1/2 and protein kinase C (PKC) phosphorylation, associated with degranulation processes, as well as Extracellular Signal-Related Kinases (ERK) 1/2, JNK, p38, and Akt phosphorylation, associated with cytokine expression [83]. Upon identification of major components of the extracts, arctigenin, a major compound (Fig. 4A), was elevated sixfold in F-AFE compared to AFE, whereas arctiin, an arctigenin glycoside, was decreased in F-AFE [83]. F-AFE- containing anti-allergic compounds, like arctigenin, inhibits the IgE-mediated allergic pathway.

Another study confirmed the anti-IgE effects of arctigenin, whereby arctigenin (10^−6^, 10^−5^, and 10^−4^ mol/L) inhibited β-hexosaminidase release, histamine release, and IL-4 production of RBL-2H3 cells with no evident cytotoxicity [84]. Effects on IL-2 and IFN-γ without cytotoxicity were also demonstrated upon arctigenin treatment of primary mouse spleen lymphocytes [84]. In vivo confirmation of the anti-allergic effects of arctigenin were demonstrated [84]. Kee and Hong also investigated these effects, confirming arctigenin (2, 10, and 50 μM) reductions on histamine, RNA expression of chemokines macrophage inflammatory protein (MIP)-1α, MIP-1β, macrophage inflammatory protein (MCP-1), and Regulated Upon Activation, Normally T-Expressed, And Presumably Secreted (RANTES) and pro-inflammatory cytokines, including IL-1β, IL-6, IL-8, and TNF-α, and protein expression of p-ERK/ERK, p-JNK/JNK, p-p38/p38 and NFκB phosphorylation in HMC-1 mast cells [85]. Receptor-interacting protein kinase (RIP2)/CASP-1, crucial to NF-κB activation, was inhibited by arctigenin treatment. In vivo IgE-mediated PCA and induced anaphylaxis were also reduced upon arctigenin treatment in a dose-dependent manner (10–50 mg/kg) [85]. Mortality of the high dose arctigenin-treated group (50 mg/kg) was 22.5%, compared to AFE and Arctii fructus water extract (AFW) groups (100 mg/kg), which were 20% and 42.5%, respectively.

### Food allergy

An independent screen of 300 medicinal herbs identified *Arctium lappa* as an effective anti-IgE natural product. The principal compounds isolated were arctigenin and arctiin, both of which markedly suppressed IgE production by U266 cells in vitro, with arctigenin showing the strongest anti-IgE activity (IC_50_ = 5.09 μg/mL) [76]. In vivo, arctigenin (0.4 mg/mouse per day) reduced peanut sIgE and histamine levels and also blocked hypothermia in a PNA murine model [76]. In vivo results coincided with the reduction of IgE production (Fig. 4B) and Th2 cytokine IL-5 and IL-13 expression by stimulated PBMCs from food-allergic patients [76]. Analysis of differentially expressed genes upon arctigenin treatment highlighted down-regulation in cell division and cell-cycle related genes, including Ubiquitin-Conjugating Enzyme E2 C (UBE2C) and Marker Of Proliferation Ki-67 (MKI67), and up-regulation of anti-inflammatory factors, including BCL6 (Fig. 4C) [76]. Notably, this group also determined that one day treatment at 15× the therapeutic dose or a 14 day treatment of 8× the therapeutic dose, all hematology testing was normal and therefore deemed safe. Arctigenin may have a potential role in treating IgE-mediated food and other allergic diseases.

### Pharmacokinetics and bioavailability

The pharmacokinetic and bioavailability profile of arctigenin is well characterized. Arctigenin is generally treated as a proxy for *A. lappa* lignan exposure. The oral administration of *A. lappa* extracts show that arctiin is rapidly hydrozlyed to arctigenin by the intestinal microbiota, and hence is the primary circulating form [86]. Rat PK studies report low-to-moderate absolute oral bioavailability, generally in the ~ 5–15% range, depending on dose and formulation, In rats and beagle dogs, arctigenin exhibited a strong absorption capacity in both rats and beagle dogs (absorption rate < 1 h), a high absorption degree (absolute bioavailability > 100%), and a strong elimination ability (half life (t1/2) < 2 h) through metabolism [87]. Their results also indicated that the distribution of arctigenin in rat tissues is rapid (2.5 h to reach the peak) and wide, detectable in almost all tissues and organs) [87]. In another study, showed that arctigenin exhibited a fast absorption phase and lasting elimination phase [88]. Normal rats administered oral arctigenin (200 mg/kg) demonstrated a better pharmacokinetic profile than those administered intravenously (10 mg/kg), including (oral vs. intravenous) Tmax (0.072 vs. 0.017 h), Cmax (127.43 vs. 527.09), t1/2 (6.91 vs. 10.46 h), and clearance (0.318 vs. 0.0084) [88]. Formulations strategies including liposome and hydrogel delivery systems have demonstrated significant improvements in arctigenin exposure in vivo, potentially improving oral delivery despite the inherent absorption and metabolic limitations [89, 90]. Further, mixing arcitgenin with other bioactive compounds has demonstrated a synergistic effect in bioavailability, and hence efficacy, in cancer models, including green tea, curcumin, and quercetin in combination [91, 92]. A Phase I trial of GBS-01, an extract from *A. lappa*, is an orally administered drug rich in arctigenin that showed high arcitgenin bioavailability, while arctiin was not detected in the pharmacokinetic profile [82]. The recommended dose of GBS‐01 was therefore determined to be 4 g of burdock fruit extract, as no dose-limiting toxicity were observed at any dose levels tested. Arctigenin is overall safe with many modalities available to further enhance its bioavailability and pharmacokinetic profile.

## ASHMI for IgE-mediated allergic diseases

MSSM-002 is a TCM herbal formulation composed of 14 herbs, including zi su zi (*Perilla fructus,* 9 g), ting li zi (*Descurainiae semen,* 9 g), xing ren (*Prunus armeniaca,* 9 g), huang qin (*Scutellariae radix,* 9 g), ku shen (*Sophora flavescens,* 9 g), dang gui (*Angelica sinesis radix,* 9 g), bai shao (*Paeoniae radix alba,* 9 g), ge gen (*Puerariae lobatae radix,* 9 g), jie geng (*Platycodonis radix,* 9 g), gan ca*o* (*Glycyrrhiza radix et rhizoma,* 6 g), *da zao* (*Jujube fructus,* 6 g), sheng jiang (*Zingiberis rhizome recens,* 6 g), *zhen zhu mu* (*Margaritifera concha,* 9 g) prepared by boiling, and then adding 150 mg of lyophilized ling zhi (*Ganoderma,* 6 g). It was prepared based on an empiric prescription, *Ja Wai San Zi Tang*, used in Bejing for childhood asthma and bronchitis treatment for coughing, wheezing, and chest tightness. The simplified anti-asthma herbal medicine intervention (ASHMI) formula consists of three of these herbs, including ling zhi, ku sheng, and gen cao. These herbs are thought to have immune regulation, anti-inflammation, and corticosteroid properties, respectively. HPLC analysis of ASHMI and its individual herbs and extracts have been reported for standardization according to guidelines issued by the US Food and Drug Administration Guidance for Industry Botanical Drug Products,

### Asthma

Li et al. investigated Chinese herbal formula MSSM-002 in a conalbumin-induced murine model of allergic asthma. MSSM-002 treatment eliminated clinical indicators of allergic asthma, including airway hyperreactivity and the total number of cells and percentage of eosinophils in the BALF [93]. Reduced inflammation, mucus production, and mitigated sIgE, IL-4, IL-5, and IL-13 levels, with no effects on IgG2a and IFN-γ were observed [93]. To determine the mechanisms underlying these effects, Th2 polarized splenocytes (Th2-SPCs) and Th2 cloned cells (D10) from mice were treated with MSSM-002, and demonstrated significantly decreased proliferation, IL-4 and IL-5 secretion, and increased IFN-γ production [94]. MSSM-002 reduced GATA-3 mRNA and protein expression and the binding to the IL-4 gene promoter in D10 cells [94]. Authors conclude that in contrast to dexamethasone that suppresses T cells overall, MSSM-002 is Th2-specific, at least partially, through GATA-3 regulation. This group also demonstrated in human intestinal mucosal Th2-like cell lines from patients with cow milk allergy, that MSSM-002 (50 µg/mL) reduced proliferative responses and IL-4, IL-5, and IL-13 secretion, with no effect on IFN-γ production [95].

The actions of the individual herbs of MSSM-002 and TCM formulation concepts enabled Wen et al. to develop a simplified anti-asthma herbal medicine intervention, ASHMI [96]. This includes only 3 of the original 14 herbs of MSSM-002, ling zhi (*Ganoderma lucidium*, 20 g), ku shen (*Sophora flavescens,* 9 g), and gan cao (*Glycyrrhiza uralensis,* 3 g). Systems pharmacology in silico analysis of ASHMI evaluated the three herbs and narrowed down active compounds with high oral bioavailability and drug-likeness, with potentially overlapping pharmacological effects [97]. It was revealed that the three herbs share common targets that include pathological processes of asthma, inflammation, and immune regulation, including TNF signaling, Phosphoinositide 3-Kinase/AKT (Protein Kinase C; PKC) (PI3K-Akt) signaling, and NF-κB signaling, to name a few [97].

**Fig. 5:**
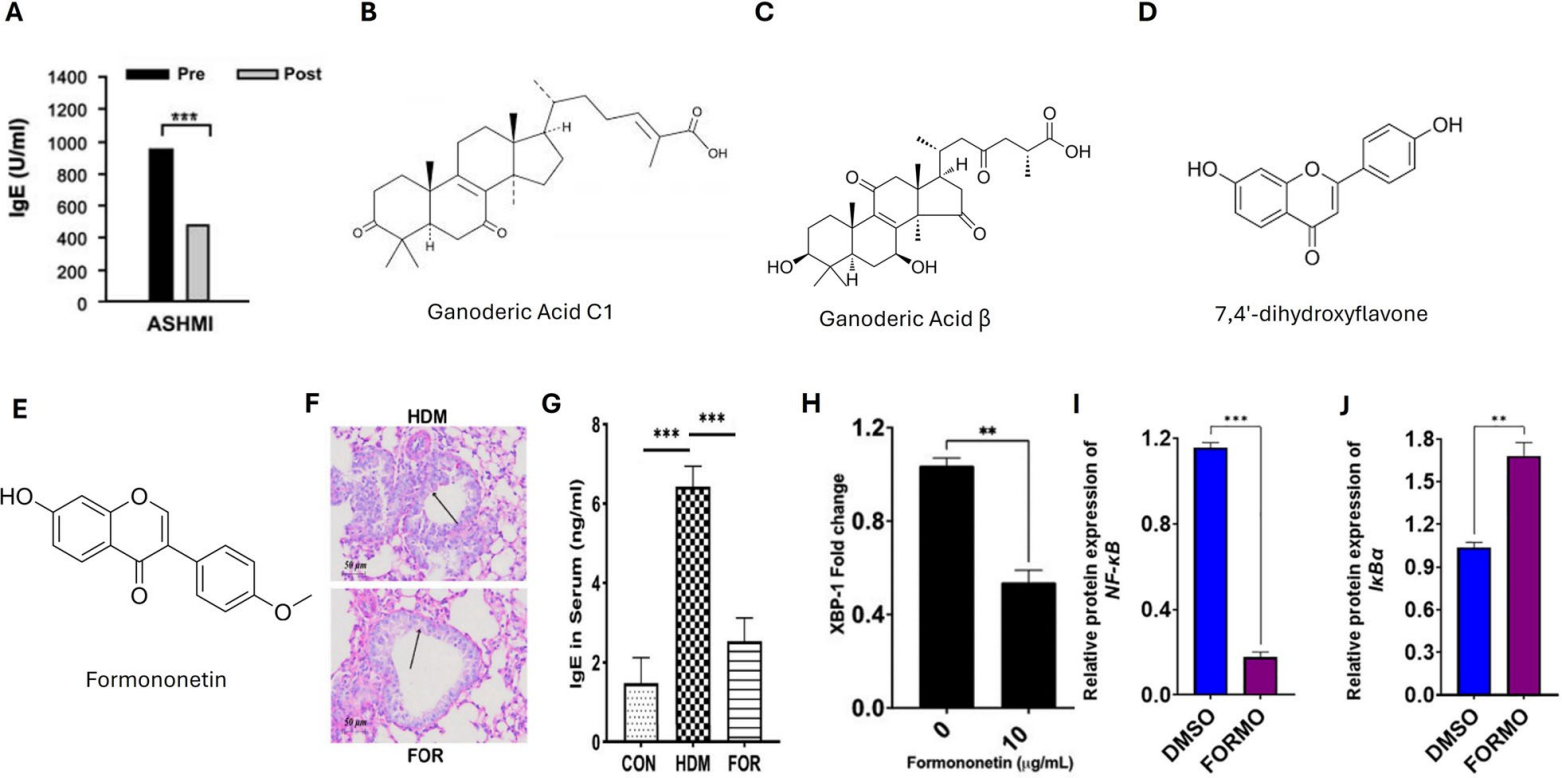
The effect of ASHMI and isolate, formononetin, on allergic asthma. **A** Four weeks of ASHMI-treatment reduced serum IgE levels in adult patients with moderate-severe allergic asthma [96]. **B** Ganoderic Acid C1 structure. **C** Ganoderic Acid β structure. **D** 7,4′-dihydroxyflavone structure. **E** Formononetin structure. **F** Formononetin (FOR) alleviated HDM-induced inflammation evident by PAS staining of lung sections (200×). Light purple area demonstrated by the black arrows (scale bar = 50 µm) [118]. **G** The concentrations of total serum IgE were reduced with FOR treatment [118]. **H**–**J** Formononetin treatment of IgE-producing myeloma cell line U266 and 10 μg/mL revealed decreases in XBP1 (**H**) and NFKB (**I**) expression while increasing IKBa (**J**) expression [121]

A double-blind, randomized trial of 91 patients with moderate-severe, persistent asthma with prednisone therapy were administered ASHMI (3.6 g) or prednisone for 4 weeks [96]. The authors discovered the ASHMI, like MSSM-002, has broad spectrum therapeutic effects on major mechanisms involved in allergic asthma, including airway hyperreactivity, pulmonary inflammation, and airway remodeling, and the downregulation of Th2 responses [96]. These therapeutic effects were evidenced by improved Forced Expiratory Volume in one second (FEV1) and Peak Expiratory Flow (PEF) values and symptoms scores, reduced inhaled β2-agonist usage, number of peripheral eosinophils, and serum IL-5, IL-13, and IgE, with increased serum cortisol and IFN-γ (Fig. 5A) [96]. ASHMI was well-tolerated with no serious adverse effects demonstrated by normal hematologyand serum chemistry [96]. Additionally, stimulated PNA human PBMCs upon treatment demonstrated reduced IL-5 and IL-13, without cytotoxicity [96, 98]. ASHMI was determined to be safe and an effective alternative to prednisone, as ASHMI has no adverse effects on adrenal function and had a beneficial effect on the Th1 and Th2 balance [96]. In the double-blind, randomized, placebo-controlled, dose-escalation phase 1 study of ASHMI (600 mg, 1200 mg, 1800 mg), no grade 3 adverse events occurred, laboratory parameters before and after treatment remained within normal ranges, and no unexpected immunological changes were detected, supporting its safety and tolerability allowing for advancement to phase 2 study [99].

Asthma occurs commonly in childhood, yet older asthmatic patients have higher risk for corticosteroid adverse effects, warranting the investigation of ASHMI on an older population. In vivo, aged mice administered ASHMI (9.5 mg/ mouse per day) saw similar results to young mice, including significantly decreased lung inflammation, airway mucous metaplasia, downregulated MUC5AC [100]. Decreased collagen production, sIgE and Th2 cytokines IL-4, IL-5, and IL-13, and increased IFN-γ were observed in isolated splenocyte and lung cultures [100]. To determine the long-term protection conferred from ASHMI (9 mg/mouse twice daily), a murine model of asthma was re-challenged 8 weeks after cessation of therapy [101]. Reduced pulmonary eosinophilic inflammation, decreased sIgE and Th2 cytokines, and increased IFN-γ levels persisted as well [101]. Neutralization of IFN-γ eliminated these effects of ASHMI, however transforming growth factor (TGF)-β neutralization did not, emphasizing IFN-γ as a critical factor for the sustained protective effects of ASHMI [101]. As maternal asthma is a risk factor for asthma development in offspring, ASHMI was investigated for its use as a preconception maternal treatment to minimize risk to offspring. Maternal therapy with ASHMI (4.5 mg/mouse twice daily) was more effective than dexamethasone at decreasing offspring susceptibility to airway disease, characterized by increased IgG2a and decreased BALF C-X-C motif chemokine ligand-1 and eotaxin-1 levels [102]. ASHMI could be a strategy to lower asthma prevalence.

ASHMI success in murine models of allergic asthma and in patient clinical trials led to the investigation into the synergistic anti-inflammatory effects of the individual herbs (ling zhi (*Ganoderma lucidium*), ku shen (*Sophora flavescens*), and gan cao (*Glycyrrhiza uralensis*)) was conducted. The constituents of ASHMI synergistically inhibited eotaxin-1 production by human lung fiborblasts and Th2 cytokines IL-4 and IL-5 by murine memory Th2 cells, as opposed to individually [103]. The IC_50_ values (mg/mL) for ASHMI, ling zhi, ku shen, and gan cao for eotaxin-1 were 38.1, 33.1, 100, and 158.5 respectively; IL-4 production were 158.5, 239.9, 446.7, and 281.8 respectively; and the IC_25_ values (25 mg/mL) for IL-5 were 30.2, 263.0, 123.2, and 100.0, respectively, The pharmacological actions of ASHMI (4.5 mg/ mouse twice daily) in early and late-phase airway responses elucidated broad effects on pathological mechanisms impeding the allergic airway response, characterized by reductions in histamine, leukotriene C4, sIgE, BALF eosinophil count, airway remodeling, and Th2 cytokine secretion by BALF and splenocyte cultures [104]. Reductions in IgE, IL-4, IL-5, IL-13 and increases in IFN-γ, IL-10, and TGF-β levels were also observed [104]. ASHMI also inhibited contraction of murine tracheal rings and increased the production of potent smooth muscle relaxer PGI(2) [104]. Acute in vivo administration and ex vivo analysis was performed to elucidate the mechanisms of ASHMI (4.5 mg/mouse twice daily) inhibition of airway hyperreactivity and tracheal ring constriction. In vivo*,* ASHMI reduced airway hyperreactivity in response to acetylcholine provocation [105]. Ex vivo*,* ASHMI significantly and dose-dependently reduced tracheal restriction to acetylcholine, which was abolished by cyclooxygenase inhibition and prostaglandin E2 receptors, EP2/EP4 receptor blockade [105]. These data together demonstrate that ASHMI had direct and acute inhibition on airway hyperreactivity in vivo and prevented acetylcholine-induced tracheal ring constriction via the EP2/EP4 receptor pathway, and hence its bronchoprotective activity [105]. Research conducted by Yang et al. aimed to investigate the individual herbs and active compounds of ASHMI that are responsible for the inhibition of airway smooth muscle contraction. The *S. flavescens* extract (45 μg/mL) specifically inhibited ASM contraction in tracheal rings of asthmatic mice [106]. The isolation of the specific compounds revealed trifolirhizin as the active component inhibiting acetylcholine mediated ASM contraction or relaxes pre-contracted ASM, independent of the β2-adrenoceptors [106]. The immunomodulatory component of ASHMI that regulates IL-5 and IL-10 production by PBMCs from asthmatic patients were assessed ex vivo*,* whereby *Sophora flacescens* fraction 2 (SF-F2) that is alkaloid rich was more effective at increasing IL-10 and fraction 4 (SF-F4) was more effective at decreasing IL-5 [107].

Neutrophil-predominant asthma has a poorer prognosis, so ASHMI and its refined formula (ASHMI(II)) were investigated for their effectiveness. Both (4.5 mg/mouse per day twice daily) markedly reduced airway hyperreactivity, mucus production, neutrophilic inflammation, TNF-α, IL-8, IL-17, eosinophilic inflammation, and Th2 responses in vivo [108]. When investigating ASHMI active compound ganoderic acid C1 (GAC1; C_30_H_42_O_7_, 514.6 g/mol; Fig. 5B) effects on murine macrophages RAW264.7 upon antigen stimulation, TNF-α and phosphorylated inhibitor of κB (IκB) decreased and histone deacetylase 2 increased [108]. The authors concluded that ASHMI and/or its GAC1 constituent may be valuable in neutrophil-predominant asthma. GAC1 is a triterpenoid with a molecular formula of C_30_H_42_O_7_ and a molecular weight of 514.6 g/mol. Investigation of chronic GAC1 treatment (20 mg/kg) demonstrated significant reductions in pulmonary inflammation and airway neutrophilia, and inhibited TNF-α, IL-4, and IL-5 levels in vivo [109]. H292 cells treated with GAC1 (40 μg/mL) showed decreased MUC5AC expression and ROS production [109]. GAC1 was also the one of 15 compounds isolated and identified from *Ganoderma lucidum*, upon being identified as the extract responsible for inhibition of TNF-α production by murine macrophages [110]. GAC1 (20 μg/mL) significantly reduced TNF-α production by both murine macrophages RAW264.7 and PBMCs from asthma patients [110]. This reduction was associated with downregulated NFκB expression and partial suppression of MAPK and AP-1 signaling, elucidating that GAC1 may have potential for treating TNF-α mediated inflammation [110]. *Gandoerma lucidum* constituent ganoderic acid β (GAB; C_30_H_44_O_6_, 500.7 g/mol; Fig. 5C) was used to treat allergy patient PBMCs and results in significant increases in IL-10 and IFN-γ levels by day 3 [111]. By Day 6, GAB inhibited IL-5 while maintaining high levels of IL-10 and IFN-γ, suppressing GATA-3 and maintaining Foxp-3 and T-bet gene expression [111]. GAB demonstrated unique beneficial cytokine modulatory effects that differentiate it from the overall suppression with dexamethasone treatment.

Investigation into ASHMI herb *Glycyrrhiza uralensis* on memory Th2 in vitro and Th2 inflammation in vivo revealed isoliquiritigenin, 7,4′-dihydroxyflavone (DHF; C_15_H_10_O_4_, 254.24 g/mol; Fig. 5D) and liquiritigenin as dose-dependent repressors of IL-4 and IL-5, with DHF being the most effective (IC_50_ (μg/mL) for IL-4 = 2.3, for IL-5 = 1.1) [112]. D10 cell proliferation and GATA-3 and IL-4 mRNA expression were repressed when treated with 6.25 μg/mL DHF, with no cytotoxicity [112]. Chronic treatment with DHF (6 μg/day) in murine models demonstrated reduced eosinophilic pulmonary inflammation, serum IgE, IL-4 and IL-13 levels and an increase in IFN-γ production in cell cultures from the lung in response to antigen stimulation [112]. DHF also demonstrated inhibition ex vivo, including eotaxin, IgE, and Th2 cytokine production, including TNF-α, IL-1β, IL-6, and IL-8 [113, 114]. Further identification and isolation of ASHMI active components may provide novel approaches to treatment of allergic diseases.

### Pharmacokinetics and bioavailability

As with FAHF-2 and its derivates, and the herbs *A. lappa* and *R. cordifolia*, the full formulation has not been assessed for pharmacokinetics and bioavailability. While ASHMI has human clinical and phase I safety data as a formula and HPLC fingerprints, what gets absorbed is not reflected in the literature. Accordingly, PK context is typically inferred from well-characterized marker constituents and/or component-herb extracts (ganoderic acid C1, isoliquiritigenin, and DHF), recognizing that multi-herb combinations may alter absorption and metabolism. Ganoderic acid C1 was assessed among other ganoderic acids in rat plasma and demonstrated a swift metabolism in systemic circulation and high distribution across nearly all tissues, with t1/2 of 1.24 h, Tmax of 0.30 h, and Cmax of 643.13 ng/mL following oral administration of Ganoderma lucidum spore powder [115]. Ganoderic acid C1 has also demonstrated satisfactory bioavailability when administered orally [109]. A novel isoliquiritigenin loaded self-nanoemulsifying drug delivery system was developed for isoliquiritigenin and demonstrated improved bioavailability in vivo 3.92 × , mainly attributed to the poor absorption, low intestinal permeability and rapid elimination of free isoliquiritigenin [116]. Pharmacokinetic profile improved (free vs. nonoemulsified) Cmax (0.37 vs. 1.17) and Tmax (0.50 vs. 0.67 h). Further, its anti-asthma effects were also improved, evidence by improved asthma-associated inflammation, including eosinophil production, OVA-specific IgE, IL-4, IL-5, and IFN-γ at 10 mg/kg compared to the group that received 20 mg/kg of free isoliquiritigenin [116].

## Formononetin for IgE-mediated allergic diseases

Formononetin is a naturally occurring isoflavone found in various plants. It has a molecular weight of 268.3 g/mol and a molecular formula of C_16_H_12_O_4_ (Fig. 5E). This phytoestrogen exhibits a broad range of physiological effects including the potential for treatment and prevention of diseases including cancer. Beyond its anti-neoplastic effects, formononetin has demonstrated anti-inflammatory, antioxidant, anti-microbial, and antiviral activity. Formononetin has demonstrated a good safety profile [117].

### Asthma and allergic rhinitis

Formononetin was investigated for its mechanism of action on airway inflammation and epithelial barrier repair in a HDM-induced asthmatic mouse model. Formononetin treatment (25 mg/kg per day) significantly decreased levels of Periodic Acid-Schiff (PAS) staining in the lungs (Fig. 5F), IgE in serum (Fig. 5G) and IL-4, IL-6, IL-10, IL-17A in BALF [118]. Decreased serum levels of alanine transaminase, aspartate transaminase, creatine, uric acid, and lactate dehydrogenase activity were observed [118]. In vitro assessments demonstrated positive effects on bronchial epithelial barrier repair including increased proliferation and migration although averting apoptosis via dampening of the Bax/Bcl2 ratio [118]. Both in vitro and in vivo treatment significantly inhibited TLR-4 and elucidated to the role of estrogen receptor 1 (ESR1)/ NLR family, pyrin domain-containing protein 3 (NLRP3)/CASP-1 signaling [118]. Additionally, in an in vitro allergic rhinitis airway epithelial model system of human nasal epithelial cells, IL-13 stimulation in the presence of formononetin treatment (10 μM) suppressed upregulated proinflammatory cytokines and mucus formation, potentially though the sirtuin (SIRT1)/Nuclear factor erythroid 2- related factor 2 (NRF2) signaling pathway [119].

### Food allergy

Yang et al. sought to identify the compounds of ASHMI responsible for IgE inhibition and their mechanism of action, separating and identifying formononetin in *Sophorae flavescentis* [120]*.* Formononetin significantly and dose-dependently (0.15–20 μg/mL; IC_50_ = 0.16 μg/mL) decreased IgE production by IgE-producing myeloma cells U266 without causing cytotoxicity [120]. This reduction in IgE was concurrent with the decreased expression of ER-stress transcription factor XBP1 (Fig. 5H), pertinent to antibody production, and the IgE heavy chain gene with formononetin treatment (10 μg/mL) [120]. To extend this data, investigation of the effects of formononetin on IgE production by PBMCs of food-allergic patients in vitro was performed [121]. In a noncytotoxic manner, formononetin decreased IgE expression by PBMCs dose-dependently (20, 10, 5, and 2.5 µg/mL) [121]. Regulation of genes including NFKBIA, TP53, BCL2, Bruton tyrosine kinase (BTK), STAT3, CCND1, STAT1, and NFKB1 were observed by formononetin treatment (10 μg/mL). Confirmed by qPCR in IgE-producing U266 cells, formononetin could act through biological processes including B cell proliferation, differentiation, immune response, and activation processes (Fig. 5I, J) [121]. The authors assessed the effect of formononetin on preventing mast cell degranulation in rat basophilic leukemia RBL-2H3 and human basophilic chronic myelogenous leukemia KU812 cells and saw reduced degranulation without any evident cytotoxicity [121]. Formononetin treatment dose-dependently reduced mast cell degranulation, histamine release, and inflammatory cytokine expression in RBL-2H3 cells and mouse bone marrow derived mast cells, reducing IgE-induced MAPK and NF-κB expression in vitro [122].

### Atopic dermatitis

Treatment of an atopic dermatitis murine model (10 mg/kg) and a human keratinocyte cell line derived from adult human skin (10 μM) with formononetin exhibited decreased Thymic Stromal Lymphopoietin (TSLP)/IL-33 levels with increased E-cadherin levels, highlighting its protective effects in allergic diseases [123]. These Th2-mediated AD mice demonstrated improved ear skin epithelial cell integrity as well [123]. A study of the effects of formononetin on fluorescein isothiocyanate-induced AD murine model (10 mg/kg) and the same human keratinocyte cell line (10 μM) confirmed reduced TSLP levels and upregulated TNF-α induced protein 3 (TNFAIP3/A20) by G protein-coupled estrogen receptor (GPER) activation [124]. Human keratinocytes in another study upon formononetin treatment demonstrated prevention of filaggrin and loricrin expression reductions with TNF-α/IFN-γ stimulation [125]. Briefly, confirming the protective effects described above -but an an IgE-independent pseudoallergic manner- in a 2,4 Dinitrobenzene (DNCB)-induced atopic dermatitis murine model, formononetin treatment (25, 50 mg/kg) reduced skin lesions, swelling, ear thickness, and mast cell infiltration [125]. This coincided with in vitro inhibition of RBL-2H3 mast cell degranulation upon formononetin treatment (10, 20, 40 μM) with suppression of morphological changes and cytoskeletal decomposition, mediated by NF-κB signaling. Primary bone marrow derived mast cells treated with up to 100 μM of formonetin showed no cytotoxicity, but reduction in degranulation (IC_50_ = 50.24 μM) and TNF-α and IL-13 mRNA expression (10, 20, 40 μM).

### Pharmacokinetics and bioavailability

Most human studies of isoflavones and flavonoids have been unsuccessful, which is mainly due to the very poor oral bioavailability. Formononetin oral bioavailability in rats was shown to be a moderate 21.8%, much higher than its glycoside ononin (7.3%) following a 20 mg/kg treatment [126]. Formononetin was rapidly absorbed and peaked at 30 min, which was followed by a quick elimination phase with a t1/2 of 2.1 h and was absorbed in all gastrointestinal segments [126]. The authors suggested that given previous research that formononetin displays slower glucuronidation in intestinal and hepatic microsomes and higher gut permeability [127], formononetin bioavailability have benefited from its structural methylation status making it more liposoluble and improving permeability [126]. They further characterized formononetin absorption, noting there was no significant difference between permeability in the A to B direction and that in the B to A direction, and transportation of formononetin across intestinal epithelial cells was mainly through passive diffusion [126]. Improvement to formononetin’s bioavailability have been sought out through advanced delivery systems including nanoparticles, phospholipid complexes, and self-nanoemulsifying systems, and have enhanced its poor solubility and absorption [128–131].

## Apigenin for IgE-mediated allergic diseases

**Fig. 6:**
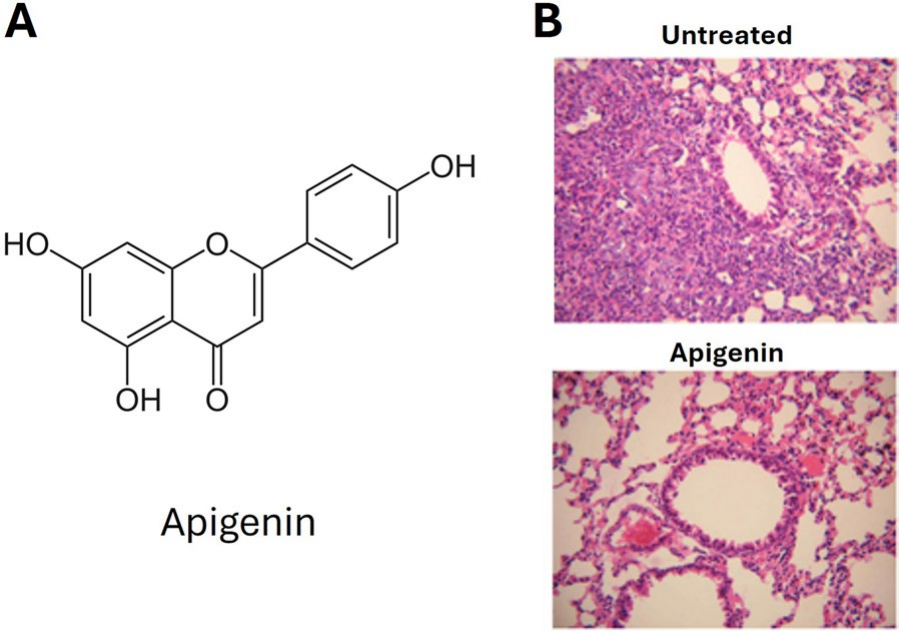
The effect of apigenin on allergic asthma. **A** Structure of apigenin. **B** Pathological changes of lung tissues observed by HE staining (Light microscopy, ×200), top is untreated, and bottom is apigenin treated (5 mg/kg). Untreated tissues demonstrated marked infiltration of inflammatory cells into perivascular and connective tissues, compared with the apigenin-treated tissue [137]

Apigenin (4′,5,7-trihydroxyflavone), is a natural flavonoid with good anti-inflammatory and antioxidant activity in various diseases. It is found in many plants and is the aglycone of several naturally occurring glycosides. It has a molecular weight of 270.2 g/mol and a molecular formula of C_15_H_10_O_5_ (Fig. 6A). Many studies have demonstrated that apigenin is non-toxic and non-mutagenic [132].

### Food allergy

The flowers of *Impatiens textori* were investigated for their anti-allergic properties, as the 35% ethanol extract demonstrated a decrease in blood pressure and exogenous platelet activating factor, which are hallmarks of allergy-mediated anaphylaxis, inflammation, and pruritus [133]. The principal compounds from *Impatiens textori* that were compared to the total extract included apigenin [133]. The authors induced food allergy in a mouse model by intraperitoneal sensitization of hen egg-white lysozyme and challenged for IgE-dependent allergic reaction assessment through tail vein intravenous injection of hen egg-white lysozyme [133]. The decrease in blood pressure induced by compound 48/80 or by antigen, the decrease in blood flow induced by antigen, and the compound 48/80-induced increase in scratching behavior were all ameliorated by the total plant extract [133]. Apigenin (20 mg/kg) was also able to prevent the reduction in blood pressure and scratching behavior caused by anaphylaxis induced by antigen and Compound 48/80, however did not significantly affect blood flow [133]. The whole extract and apigenin were also able to prevent platelet activating factor and serotonin hydrochloride-induced scratching [133]. Therefore, the flowers of Impatiens textori, and potentially its principal compound apigenin, may serve as anti-anaphylactic and anti-pruritic agents.

### Atopic dermatitis

An atopic dermatitis CD48/80-induced itch murine model treated with apigenin (75, 150 mg/kg) saw reduced the number of scratches and inhibited the infiltration of polymorphonuclear lymphocytes [134]. In murine macrophages, apigenin treatment (100 μM) inhibited NO production, expression of cytokines IL-1β, IL-6, COX-2 and iNOS, and MAPK signaling molecules, including ERK and JNK in a noncytotoxic manner [135]. They also found that in human keratinocytes, apigenin treatment (20 μM) had no cytotoxic effects and induced filaggrin, loricrin, aquaporin-3, hyaluronic acid (HA) and HA synthase (HAS)-1, -2, and -3, which constitute physical barrier of the skin [135]. Expression of antimicrobial peptides constituting chemical skin barriers including human β -defensin (HBD)-1, 2, -3, and cathelicidin were also increased [135]. Apigenin proved to suppress the inflammation and allergic responses elicited by macrophages and mast cells, while also improving the skin barrier, and hence may be a treatment option for atopic dermatitis.

### Asthma and allergic rhinitis

Apigenin demonstrated the ability to attenuate allergen-induced airway inflammation in a murine model of OVA-induced asthma, decreasing the degree of inflammatory cell infiltration, airway hyperresponsiveness, total IgE levels, and demonstrating a Th1 profile [136]. The reduced infiltration of inflammatory cells into perivascular and connective tissues was also demonstrated in another study with apigenin treatment (5 mg/kg, 10 mg/kg) (Fig. 6B) [137]. This reduced inflammation was characterized by decreased airway resistance, eosinophil count, eosinophilia, Th17 cells, and Retinoic acid receptor-related orphan receptor (ROR)γt protein and restored IL-6, TNF-α and IL-17A levels [137]. These were further confirmed in another study of murine eosinophilic asthma, whereby apigenin (25 mg/kg) reduced airway hyperresponsiveness, airway inflammation, and Th2 cytokines IL-4, IL-5, IL-13, eosinophil derived neurotoxin, and eosinophil peroxidase [138]. This Th2 response was characterized by the reductions of total cells, macrophages, eosinophils, peri bronchial and perivascular inflammatory cells, mucus production and aryl hydrocarbon receptor expression upon MnBP treatment [138]. Here, murine splenic MNCs demonstrated reduced IL-4, IL-5, and IL-13 expression as well, in vitro upon 1 μM apigenin treatment [138]. Human Jurkat T cells, eosinophilic leukemia cells, and lung epithelial cells were also utilized by this group to assess the effects of apigenin (1 μM). Jurkat T cells demonstrated reduced phorbol 12-myristate 13-acetate (PMA)-activated increases in IL-4, IL-5, and IL-13 without cytotoxicity [138]. Butyric acid-stimulated eosinophilic leukemia cells showed reduced increases in IL-5 and eosinophilic cationic protein (ECP) [138]. Lastly, lung epithelial cells had decreased aryl hydrocarbon receptor (AhR) and nuclear translocator of AhR (ARNT) expression and enhanced AhR-silenced reductions in IL-25 and IL-33 [138].

The determination of a mechanism of action of apigenin (10, 20 mg/kg) was performed in another study that confirmed reduced inflammation, mucus secretion evident by PAS staining, and airway remodeling evident by collagen volume that was characterized by asthma-related inflammatory cytokine and apoptosis reduction in BALF, including IgE, IL-4, IL-5, IL-13, and IL-17 [139]. Transcriptomic analysis demonstrated anti-inflammatory and anti-apoptotic effects were through MAPK pathway inhibition, showing inhibited phosphorylation of ERKs, JNKs, and p38 MAPKs with downstream upregulation of BCL2 and downregulation of Bax, CASP-3 and cytochrome (cyt) C [139]. In vitro, apigenin treatment (10, 20 μM) improved cell viability, inhibited ROS production, and reversed stimulation-induced decreases in mitochondrial membrane potential and apoptosis in airway epithelial cells via MAPK signaling of human HDM- stimulated bronchial epithelial cells [139]. This was evident by the inhibition of JNK, ERK and p38 phosphorylation and downregulation of cyt- C, Bax, cleaved CASP-3 and upregulation of BCL2 [139]. Monocyte-derived chemokine (MDC) also plays a pivotal role in Th2 cell recruitment during the allergic inflammation process, and apigenin inhibited its production by THP-1 monocytes [140]. Th1 chemokine interferon inducible protein 10 (IP-10) levels have been shown to be elevated in BAF of asthmatic children, and apigenin also reduced its production by monocytes, coinciding with inhibited phosphorylation of JNK and MAPK pathways [140].

Ex vivo bronchial biopsies derived from asthma patients and human lung fibroblasts culture in vitro demonstrated apigenin (10, 20 μM) attenuates TGF- β induced fibroblast-to-myofibroblast transition, in a non-cytotoxic and non-cytostatic manner, potentially correlated with the inhibition of α-smooth muscle actin and tenascin C expression [141]. A possible interference of TGFβ-1 induced myofibroblasts and potential bronchial wall remodeling during the asthmatic process was suggested [141]. In vivo OVA-induced allergic rhinitis models treated with apigenin demonstrated reduced sIgE, IgG1, IgG2A, β -hexosaminidase, histamine, and ECP levels in mouse serum [142]. Alleviated nasal symptoms and nasal eosinophilic infiltration and decreased Th2 cytokine and transcription factor levels, with increased Th1 cytokines and transcription factors, promoting the Th1/Th2 ratio were evident [142]. Apigenin blocked lipopolysaccharide (LPS)-induced cytotoxicity, apoptosis, and inflammatory cytokine secretion by suppression of the TLR4/MyD88/NF-κB pathway [142]. Apigenin also decreased IL-4 production by T cells, highlighting its potential for ameliorating allergic symptoms or prevention of allergic disease onset [143]. Apigenin inhibited highly purified peripheral human basophils, which play a role in IgE-dependent allergic inflammation through the release of histamine and other inflammatory mediators, from producing IL-4 and IL-13 [143]. In vitro, apigenin inhibited β -hexosaminidase and histamine release in a noncytotoxic manner [134]. Apigenin (10, 30 μM) modulates IL-31 expression and release in stimulated human mast cells, with reductions in MAPK and NF-κB p65 phosphorylation activation evident by suppression of JNK, ERK, and p38 activation, and expression of PKC, IκB kinase (IKK), and IκB [134]. In vivo, apigenin treatment prevented infiltration and degranulation of mast cells, while suppressing IL-31 in the skin [134]. In RBL-2H3 cells, apigenin treatment (30 μM) also inhibited these signaling molecules, and the expression of FcεR1α and cytokines TNF-α, IL-4, IL-5, IL-6, IL-13, and COX-2 [135]. Inhibition of the phosphorylation of Lyn, Syk kinase, and PLC-ϒ, which are essential for activation, and inhibition of ERK and JNK activation was also observed [135]. Apigenin is a promising strategy for mitigation of the inflammatory response of allergic rhinitis.

### Pharmacokinetics and bioavailability

Apigenin has high permeability and low water solubility, making it readily penetrable to the host plasma membrane [144]. It is lipophilic and the acidic environment of the gastrointestinal tract can deactivate it, leading to its reduced bioavailability [144]. The location and degree of glycosylation greatly affect its absorption in the gastrointestinal tract, and it has been demonstrated that 5–10% of apigenin can be absorbed following polyphenol consumption, however reports on absorption rate are not consistent [144–146]. Regarding its metabolism, two phases are involved, first in the liver and then in intestinal and hepatic circulation [147, 148]. It is quickly and widely distributed throughout human organ and tissues, with considerably high levels in the blood and liver [144, 149, 150]. However, its distribution and bioavailability are affected by formation of conjugates by methylation, sulfation, and glucuronidation [144, 149]. For example, Borges et al. assessed bioavailability of apigenin and its O-glycosides in healthy male adults, administered as an apigenin capsule, parsley, as a drink or dried with yogurt, or chamomile tea (191, 188, 14.5 μmol, respectively) which demonstrated poor absorption of apigenin and intra- and inter- individual variations on level of its metabolite secretions [151]. In an in vitro model system, a cumulative 26% of the supplemented apigenin was absorbed by intestinal epithelium over 240 min and extensive phase I and II metabolism occurred yielding apigenin metabolites, mainly apigenin-7-sulfate, attributing to apigenin’s low overall bioavailability [152]. To enhance its bioavailability, drug delivery systems including emulsions, nanostructured lipids, hydrogels, and liposomes have been recommended and employed with some success including improved chemical stability, better penetrability, and enhanced bioactivity [144, 153–156].

## Luteolin for IgE-Mediated Allergic Diseases

**Fig. 7:**
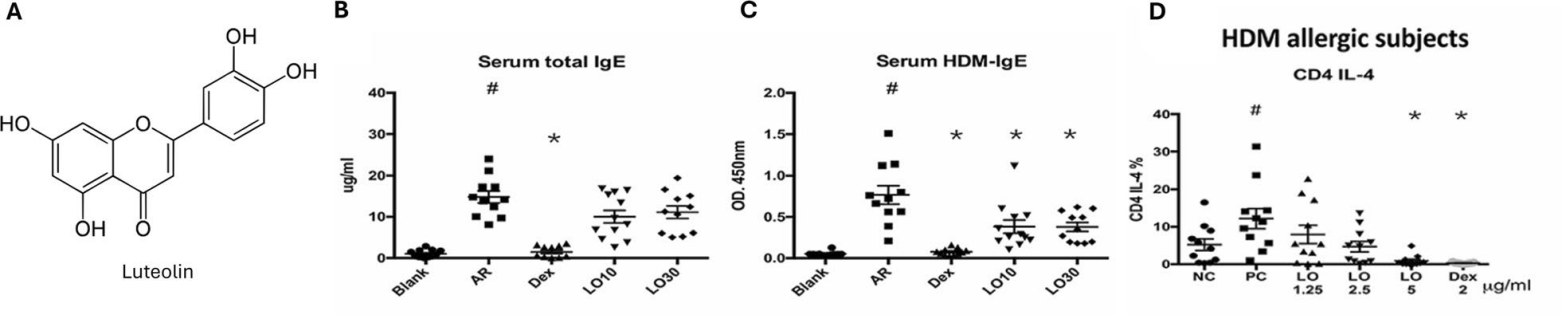
The effect of luteolin on allergic asthma. **A** Structure of luteolin. Luteolin treatment (10 mg/kg and 30 mg/kg) reduced, **B** total serum IgE and **C** HDM specific IgE. Treatment of luteolin (5 μg/mL) significantly reduced, **D** CD4+IL-4 secreting cells percentage. #*p* < 0.05 versus blank group; *p < 0.05 versus AR group [162]

Luteolin (3′,4′,5,7-Tetrahydroxyflavone (THF); Fig. 7A) has a molecular formula C_15_H_10_O_6_ and a molecular weight of 286.24 g/mol. Luteolin is a naturally-occurring flavonoid identified in hundreds of plant species [157]. Luteolin has various functions as an antioxidant, anti-tumor, anti-diabetic, and anti-inflammatory with great therapeutic potential in obesity, Alzheimer’s diseases, and Parkinson’s disease [157]. Using whole plants in Chinese medicine treatment and identifying their active compounds have highlighted luteolin as a potential therapeutic across various allergic diseases.

### Food allergy

The study that investigated the flowers of *Impatiens textori* for their anti-allergic properties also showed that luteolin was able to prevent the reduction in blood pressure caused by anaphylaxis induced by antigen and Compound 48/80, however luteolin (20 mg/kg) treatment did not significantly affect blood flow or scratching behavior [133]. Further investigation of Luteolin could demonstrate it is beneficial as an anti-anaphylactic compound.

### Asthma and allergic rhinitis

*Gerberae Pilosellodis Herba* is a commonly used TCM for allergic asthma. Elucidation of its pharmacological mechanism through molecular docking suggested luteolin as one of three main active components closely related to asthma, specifically the IL-17 inflammatory pathway [158]. In another study, luteolin (1 mg/kg) was assessed in vivo in OVA-sensitized BALB/c mice [158]. Following luteolin treatment modulated airway bronchoconstriction and bronchial hyperreaction and immunologically reduced OVA-specific IgE levels, IL-4, and IL-5 levels, and increased IFN-γ levels in the BALF [158]. The authors suggested that luteolin could serve as an anti-asthma therapy.

Another study showed similar results in their OVA-induced BALB/c asthma model, whereby luteolin (20 mg/kg) inhibited airway responsiveness and inflammation, with significant reductions in OVA-specific IgE in the BALF [159]. The total numbers of CD19+ B cells, CD4+ T cells, CD3-CCR3+, and CD11b+Gr-1+ cells in the lungs were significantly lowered with luteolin treatment [159]. The number of eosinophils and the percentage of CD3ε-CCR3+ and CD3ε+CCR3+ cells in the BALF were significantly lowered in mice sensitized and treated with luteolin as well [159]. This corresponded with reduced eotaxin-2, CCR3, IL-13 and TNF-α expression in the lung tissue, while Foxp3, IL-10 and TGF-β1 levels were significantly upregulated [159]. In the BALF, IL-4, IL-5, and IL-13 levels were also reduced and IFN-γ increased with luteolin treatment [159]. Evaluation of Tregs in vitro highlighted that CD25 expression was upregulated in luteolin-treated anti-CD3/CD28-activated CD4+CD25- T cells, characterized by upregulated IL-2, IL-10, and TGF-β1 mRNA expression and downregulated IL-4 and IL-13 mRNA levels [159]. Foxp3 mRNA expression was also induced, consistent with the acquisition of Treg suppressive function [159]. Therefore, the anti-asthma function of luteolin may be achieved through Treg modulation.

In agreement with the modulation of inflammation elucidated through in silico analysis, an in vivo OVA-induced BALB/C model sought to determine if the attenuation of airway inflammation was mediated by regulation of autophagy [160]. OVA-induced asthmatic mice were treated with low (10 mg/kg) or high (20 mg/kg) doses of luteolin and showed that high-dose luteolin significantly reduced airway resistance and improved lung function, with great improvement in inflammation, mucus secretion, and collagen deposition [160]. Immunologically, IL-4, IL-5, IL-13, and OVA-specific IgE were significantly reduced. Evaluation of autophagy markers in the lung tissues showed inhibition of LC3B, eosinophil main basic protein, and promotion of p62 expression [160]. Protein analysis of lung tissue showed significant increase in PI3K p85, p-mammalian target of rapamycin (mTOR) and p-Akt, and the regulator of the autophagy process, Beclin-1-PI3KC3 protein complex, highlighting the anti-autophagy role of luteolin in allergic asthma [160].

Luteolin was also assessed for its in vitro antioxidative effects as a preclinical model of asthma [161]. RAW 264.7 macrophages treated with Luteolin (50 μg/mL) showed no protective effect on the reduced-to-oxidized glutathione ratio but did interfere with heme oxygenase (HO)-1 oxidative stress protein expression [161].

The anti-allergy functions of luteolin have also been investigated in allergic rhinitis. Mice treated with low dose (10 mg/kg) or high dose (30 mg/kg) of luteolin before sensitization to HDM resulted in the decrease of sneezing, nose scratching, and HDM-specific IgE, without affecting total IgE, IgG1, or IgG2a levels (Figure 7B, C). Analysis of splenocytes demonstrated decreased CD4+IL-4+ and CD4+IL-17-secreting T cells when treated with high dose luteolin, which corresponded with the re-stimulation of splenocytes resulting in significant decreases in IL-4 expression [162]. A decrease in IL-10 and IL-17 expressions were observed in the splenocytes from the low dose luteolin group [162]. In the high dose luteolin group, there was a decrease in IL-4 expression in nasal lymphoid tissues [162]. In both high and low dose treatment groups, submucosal cellular infiltration and eosinophils in the laminar propria were significantly reduced, with reduced mucus production [162]. When assessing luteolin treatment after HDM sensitization, frequency of nose scratching and sneezing, and percentage of CD4+IL-4+ cells in splenocytes were reduced (Figure 7D) [162]. There were, however, no differences observed between the low or high dose luteolin treated groups and untreated mice regarding level of IgE, HDM-specific IgE, IgG, or IgG2a [162]. Yet, eosinophil infiltration decreased in nasal tissue upon luteolin low- and high-dose treatment [162]. In vitro, CD4+ splenocytes treated with 10 μg/mL luteolin showed significantly reduced IL-4 secreting CD4+ cells [162]. Naïve CD4+ cells from BALB/c mice spleen when pre-treated with luteolin showed decreased expression of p-STAT6 and GATA-3, reducing Th2 cell differentiation [162]. The PBMCs of allergic rhinitis patients pre-treated with luteolin (1.25, 2.5, 5 μg/mL) reduced CD4+IL-4- secreting cells at 5 μg/mL, confirming luteolin’s inhibition of Th2 cells expression [162].

### Atopic dermatitis

The Jiu-Wei-Yong-An formula is used to treat atopic dermatitis, improving skin lesions, itching, and thickening, mediated by JAK1/STAT3 and MAPK signaling. It consists of Rhizoma Smilacis Glabrae (*Smilax glabra*), Dioscorea Opposita (*Dioscorea oppositifolia*), Fructus Forsythiae (*Forsythia suspensa*) Rhizoma Dioscoreae Hypoglaucae (*Dioscorea collettii*), Radix Rehmanniae (*Rehmannia glutinosa*), Semen Plantaginis (*Plantago asiatica*), Isatis indigotica Fortune (*Isatis tinctoria*), Rhizoma Alismatis (*Alisma plantago-aquatica*) and Radix Angelicae Sinensis (*Angelica sinensis*). In silico analysis demonstrated that among its active components, luteolin has high affinity for JAK1 [163]. Similarly, a skin-related disease TCM treatment, *Stellera chamaejasme*, was investigated for its active component for atopic dermatitis treatment and in silico analysis identified luteolin [164]. In vitro, RBL-2H3 cells pre-treated with luteolin (10 μM) showed inhibition of IL-4 expression and mast cell degranulation[164]. However, luteolin was not further investigated in their SKH-1 DNCB-atopic dermatitis induced model.

### Mast cells

In TCM, asthma has been treated with Qi-Wei-Du-Qi-Wan and You-Gui-Wan, both with demonstrated mechanisms of action through mast cell modulation and identification of luteolin as a candidate active compound. You-Gui-Wan, composed of 10 medicinal materials, including Shou Ti Huang (*Rehmannia glutinosa*), Shan Yao (*Dioscorea opposita*), Shan Chu Yu (*Cornus officinalis*), Du Zhong (*Eucommia ulmoides*), Gou Qi Zi (*Lycium chinense*), Yu Si Zi (*Cuscuta australis*), Pao Fu (*Aconitum carmichaeli*), Jou Kue (*Cinnamomum cassia*), Lu Jiao (*Cervus elaphus*), and Dang Gui (*Angelica sinensis*) [165]. To understand the underlying mechanism behind You-Gui-Wan’s anti-asthma activity, in vivo intratracheal sensitization of BALB/c mice with Dermatophagoides pteronyssinus to induce chronic asthma was followed by You-Gui-Wan treatment before challenge. Treatment significantly decreased tracheal hyperreaction (dyspnea), and reduced serum total IgE and the IgG1/IgG_2A/2B_ ratio in a dose-dependent manner (0.2 g/kg, 0.5 g/kg) [165]. Evaluation of the expression of lung IL-6, IL-4, IL-13, COX-2, and TNF-α showed a significant dose-dependent decreases with You-Gui-Wan treatment (1 μg/mL, 3 μg/mL, 6 μg/mL) [165]. RBL-2H3 mast cells were selected, as they express IgE and those five genes, for stimulation and evaluation of gene expression and degranulation. You-Gui-Wan treatment significantly inhibited IL-6, TNF-α, IL-4, IL-13, COX-2, arachidonate lipoxygenase (ALOX)-4, and HDC expression in a dose- and time-dependent manner and demonstrated significant inhibition of mast cell degranulation [165]. 21 compounds were identified by mass spectrometry and ranked by their potential ability to inhibit ALOX-5 and HDC gene expression in silico, and suggested Luteolin as a strong candidate for modulation of mast cell activation. Luteolin was also implicated as an active component of Qi-Wei-Du-Qi-Wan, a TCM used for chest tightness, cough, shortness of breath, and asthma [166]. Qi-Wei-Du-Wan consists of Shou Ti Huang (*Rehmannia glutinosa*), Shan Chu Yu (*Cornus officinalis*), Shan Yao (*Dioscorea opposita*), Fu Ling (*Wolfiporia extensa*), Mou Tan Pi (*Paeonia suffruticosa*), Tse Hsieh *(Alisma plantago-aquatica*), and Wu Wei Zi (*Schisandra chinensis*) [166]. Using the same chronic asthma model described above, Qi-Wei-Du-Qi-Wan (0.17 g/kg, 0.5 g/kg) significantly decreased tracheal hyperreaction and improved lung pathology, including reduced inflammatory cell infiltration and sputum production [166]. Immunologically, Qi-Wei-Du-Qi-Wan treatment resulted in reduced total IgE and Dermatophagoides pteronyssinus-specific IgE levels and lung tissue expression of IL-12β, IFN-γ, IL-4, IL-13, MCP-1, RANTES, and eotaxin significantly decreased [166]. Treatment of stimulated RBL-2H3 cells with Qi-Wei-Du-Qi-Wan significantly reduced IL-4 and IL-13 expression and mast cell degranulation in a dose-dependent manner [166]. Luteolin at the equivalent concentration in the Qi-Wei-Du-Qi-Wan mixture (8.22 pg/mg; 0.14 nM) did not suppress mast cell degranulation [166]. However, determination of an effective dose of luteolin warrants further investigation.

Luteolin was shown to be more potent than cromolyn (disodium cromoglycate) at inhibiting histamine release from mast cells [167]. Pretreatment of human LADR mast cells with 100 mM of luteolin showed significant inhibition of histamine, tryptase, matrix metalloproteinase (MMP)-9, vascular endothelial growth factor (VEGF), IL-1B, IL-6, IL-8, and TNF [167]. The authors suggest that luteolin, especially in a liposomal form, would both increase its absorption and be advantageous to cromolyn [167]. Human umbilical cord blood- derived culture mast cells pre-treated with luteolin at 100 μmol/l inhibited the IgE-mediated release of VEGF in another study [168]. In agreement with this, mast cell IgE sensitization followed by luteolin treatment inhibited the release of histamine, leukotrienes, prostaglandin D2, and granulocyte macrophage-colony stimulating factor upon stimulation [169]. It inhibited Ca^2+^ influx, PKC translocation and activity, and activation of ERK and JNK [169].

In addition to mast cells, the effect of luteolin treatment on basophils has been investigated [170]. Purified basophils pre-incubated with luteolin strongly inhibited IL-4 synthesis (IC_50_ = 2.6 μM). At 30 μM, luteolin completely suppressed the synthesis of IL-4 and CD40 ligand expression of anti-IgE and IL-3 stimulated purified basophils and PMA-stimulated KU812 basophils [170]. At this concentration, luteolin significantly suppressed phosphorylation of c-Jun at Ser63 and Ser73 residues in nuclear extracts, with no effects on p-Syk, p-Lyn, or p38 MAPK, p44/p42 MAPK, or p54/56 SAPK/JNK in cytoplasmic, or c-Jun in nuclear fractions[170]. Therefore, luteolin acts through AP-1 transcription factor c-Jun, inhibiting AP-1 activation. In agreement with this, the study that identified apigenin as an inhibitor of IL-4 and IL-13 production by highly purified peripheral human basophils, the authors identified luteolin as a potential modulator of allergic disease onset or symptom management [143].

### Pharmacokinetics and bioavailability

Studies have revealed that luteolin has low oral bioavailability with poor stability, rapid absorption and wide distribution, and moderate rate of elimination in rats [171]. The bioavailability of total luteolin (200 mg/kg oral dose), including free and bound forms, has been reported to be 53.9% with free luteolin being the lowest (17.5%) [172, 173]. Luteolin-30-O-b-D-glucuronide was the most abundant form both in plasma and most of the tissues [172]. In another study of rats, following a 50 mg/kg dose of luteolin orally or intravenously, luteolin bioavailability was shown to be low, at 4.10%, with a rapid absorption and metabolism rate, large distribution, and high clearance, suggested to be owed to an enterohepatic recirculation [174]. Cyclodextrin complexation, nanocarriers, polymeric micelles, phospholipid complexation, and nanocrystals have been used to enhance the solubility and bioavailability of luteolin via slowing of the degradation in the blood stream and demonstrating increased dissolution and bioactivity, largely in cancer models [173, 175–182].

## Resveratrol for IgE-mediated allergic diseases

**Fig. 8:**
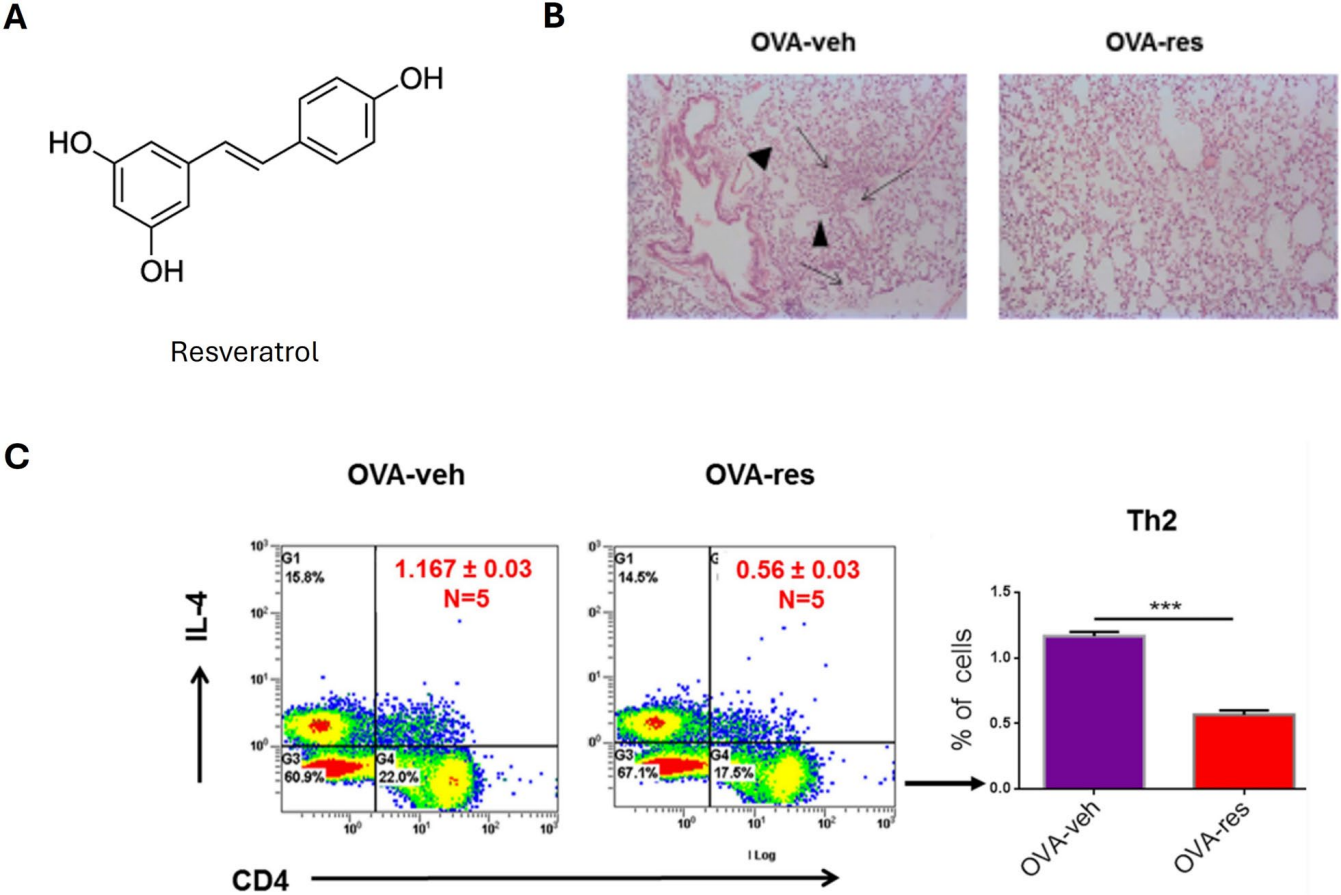
The effect of resveratrol (res) on allergic asthma. **A** Structure of resveratrol. **B** Histopathological analysis of the lungs shows significant thickening was observed in the alveolar walls and inflammatory cell infiltration as well as accumulation of edematous fluid in the alveolar spaces (arrows) and destruction of airways (arrowheads) in OVA-vehicle (veh) groups compared OVA-res groups [194]. **C** Representative flow cytometric analysis of CD4+IL4+Th2 cells shows their reduction in lungs of OVA-administered mice upon resveratrol treatment [194]

Resveratrol (3,5,4′-trihydroxy-trans-stilbene) has demonstrated anti-inflammatory, anti-neoplastic, antioxidant, cardioprotective, vasorelaxant, neuroprotective, and anti-allergic properties [183]. This natural polyphenol has been detected in over 70 plant species. Its molecular weight is 228.24 g/mol and molecular formula is C_14_H_12_O_3_ (Fig. 8A). Resveratrol is considered generally safe and well-tolerated [183].

### Food allergy

An OVA and cholera toxin-induced murine model of food allergy treated with standard diet with 0.01% resveratrol showed inhibited CT-driven mucosal sensitization to OVA, evident by reduced sIgE in serum and prevention of hypothermia [184]. IL-13 and IFN-γ levels decreased in the MLN and spleen of these mice [184]. In the derived splenocytes, resveratrol (10, 30 μM) reduced the induction of CD25+ cells, IL-2, IL-4, IL-13 and IFN-α associated with inhibition of GATA-3 and T-bet, suggested to be achieved through inhibition of dendritic cell maturation via co-stimulatory molecule CD80 and CD86 expression, and consequential early T cell activation and differentiation by repression of cyclic adenosine monophosphate (cAMP) activation of bone marrow derived dendritic cells [184]. The authors noted that this concentration is that of which can be obtained by red wine intake in humans (10 μM). In vivo, OVA-induced murine models treated with resveratrol saw suppressed clinical manifestations including diarrhea and upregulated rectal temperature, with decreased sIgE, MCP-1, and histamine [185]. Concurrently, populations of dendritic cells, B cells, and mast cells decreased, with bone-marrow derived cells ability to release β -hexosaminidase and histamine being inhibited and the alleviation of mast cell-mediated PCA reactions [185]. Resveratrol may be a prophylactic or therapeutic intervention to alleviate food allergy.

Murine OVA-induced allergic enteritis, a form of food allergy induced by oral allergens, was used and showed resveratrol (50 mg/kg) prevented mast cell degranulation and prevented increases in the mast cells in the duodenum and colon [186]. In vitro, resveratrol treatment (50 μM) of IgE/ 2,4-dinitrophenol -activated human mast cells and LPS-activated murine bone marrow derived mast cells showed reduced degranulation and expression of pro-inflammatory cytokines CCL2 and TNF-α [186]. The mechanism of mast cell inhibition by resveratrol was characterized in human (LAD2) and mouse (MC/9 and BMMC) mast cells [187]. Resveratrol (50 μM) prevented phosphorylation of ERK and FcεRI- stimulated degranulation of LAD2 [187]. In mice, resveratrol (10 μM) inhibited FcεRI expression in a concentration-dependent manner, reducing the release of TNF, without affecting phosphorylation and degranulation of SYK/ERK [187].

### Atopic march

The natural progression from atopic dermatitis in infancy to food allergies, then allergic rhinitis, then potentially asthma later in life constitutes the atopic march. In a well-established murine model of TSLP-driven atopic march, inducing atopic dermatitis and asthma, resveratrol treatment reduced airway resistance and infiltration of total cells, eosinophils, and neutrophils in both lung and ear skin tissues [188]. There was dramatically less epidermal and dermal thickness of the ears observed. In these HDM + MC903 asthma murine models of TSLP-mediated atopic march, serum inflammatory markers and NF-κB pathway-related protein phosphorylation were reduced, including TSLP, IL-4, IL-5, and IL-13, and p-p65, p65, pIκBα, and IκBα, respectively [188]. Resveratrol may protect from the TSLP-induced atopic march.

### Atopic dermatitis

The use of a canine model of atopic dermatitis whereby PBMCs were treated with resveratrol (1.5–9 μg/mL) and compared to healthy control canine PBMCs was performed. In both groups, no cytotoxicity was observed and MCP-1 and IL-6 levels were reduced [189]. The authors concluded that overall, at this concentration tested, 9 μg/mL, only a minimal effect on host defense peptides and pro-inflammatory cytokines were observed [189]. Resveratrol delivery through a nanoemulgel system (RES-NEG)-treated DNCB AD-induced mice skin showed concentration-dependent (0.5%, 0.75% and 1% w/w) improvement in the skin, as indicated by lesion severity score [190]. High mobility group box 1 (HMGB1), RAGE, TLR4, NF-κB and pNF-κB expression reduction in mouse skin tissues and the decreased expression of pro-inflammatory cytokines TSLP, IL-4, IL-6, IL-13, IL-31 and TNF-α was also observed [190]. In a HA- hydrogel containing resveratrol-loaded (10 mg) chitosan (CS) nanoparticles, release of resveratrol was observed, and it counteracted oxidative damage that was induced by ROS generation in a TNF-α/IFN-γ-treated (AD-like) human keratinocytes, without induction of cell death [191]. Pre-treatment with these resveratrol-hydrogels reduced the secretion and mRNA expression of proinflammatory cytokines IL-4, IL-5, IL-5, IL-13, IL-25, IL-33, and TSLP in these human keratinocytes in a time-dependent manner [191]. Given that basophils may be highly important in IgE-mediated very-late-phase skin inflammation, Tanka et al., assess resveratrol’s effect on human basophil activation with IgE stimulation [192]. Resveratrol treatment (100 μM) significantly suppressed histamine and leukotriene C4 (LTC_4_) release upon basophil activation [192].

### Asthma and allergic rhinitis

An HDM murine model of asthma treated with resveratrol (50 mg/kg) demonstrated reduced IL-5, IL-17, TGF-β, and TNF-α levels in BALF, coinciding with attenuated fibrotic responses including collagen deposition and airway inflammation [193]. An OVA-induced murine asthma model demonstrated that resveratrol treatment (100 mg/kg) reduced IL-5, IL-13, TGF- β in both the serum and BALF [194]. Clinically, congestion was reduced by perivascular and perialveolar inflammatory cell infiltrate and fluid extravasation that destroys alveolar walls (Fig. 8B) [194]. Decreases in CD3+CD4+, CD3+CD8+, and CD4+IL4+ cells (Fig. 8C) and increases in CD4+CD25+FOXP3+ cells were also observed [194]. In the OVA-induced asthma lung infiltrations cells, significant alterations to miRNA levels were evident, specifically miR-34a downregulation which targets FOXP3, and hence T regulatory functions [194]. In the same model of allergic rhinitis, resveratrol (200 μg or 400 μg/mouse) was confirmed to reduced symptoms including sneezing, nasal rubbing, inflammatory cytokines, and eosinophil numbers [195]. Specifically, sIgE and histamine in the serum, and sIgE, PDG2, LTC_4_, ECP, IL-4, IL-5, IL-6, IL-33, and TNF-α in the nasal lavage fluid were reduced [195]. Thioredoxin-interacting protein (TXNIP), a producer of ROS, and its associated oxidative stress pathway, including MDA and SOD were also reduced in the nasal tissue and spleen mononuclear cells, serving as a potential mechanism of resveratrol [195].

**Fig. 9:**
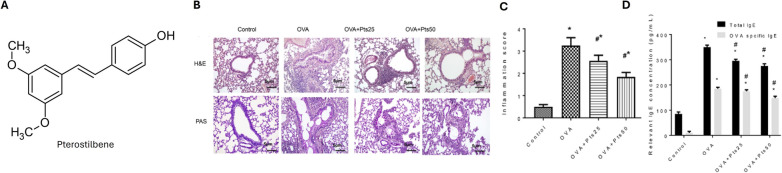
The effect of pterostilbene on allergic asthma, **A** Structure of pterostilbene. **B** Pterostilbene treatment reduced PAS+staining, indicating reduced goblet cell production and mucus secretion and **C** inflammatory cell infiltration. **D** Pterostilbele reduced total IgE and OVA specific IgE in BALF. *p < .05, compared with the Control group. #*p* < .05, compared with the OVA group [206]

**Fig. 10:**
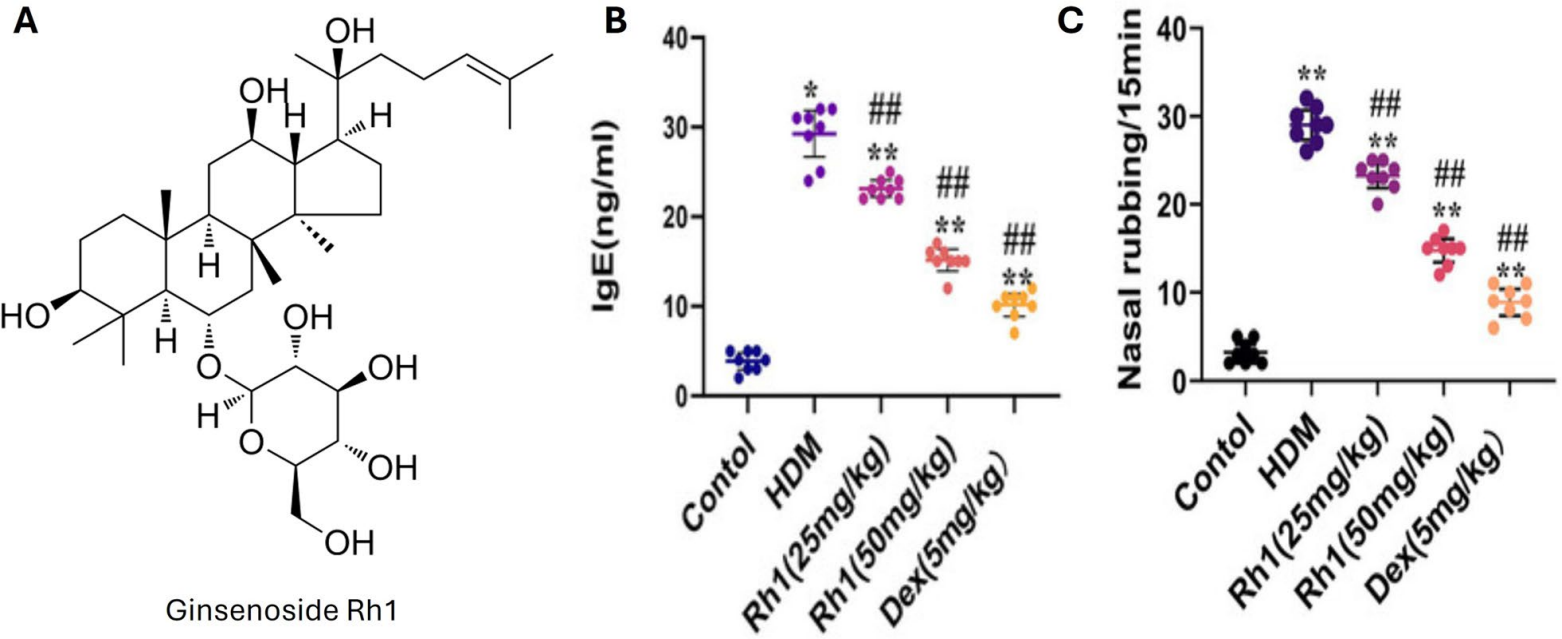
The effect of ginsenoside Rh1 on allergic rhinitis. **A** Ginsenoside Rh1 structure. **B** ELISA measurements showed ginsenoside Rh1 reduced levels of HDM-specific IgE in serum. **C** Ginsenoside Rh1 reduced the frequency of nasal rubbing observed in the mice. *p < 0.05, **p < 0.01 compared with control group. #*p* < 0.05, ##*p* < 0.01 compared with HDM-challenged group [219]

In vivo, resveratrol treatment of rats with allergic rhinitis demonstrated reductions in allergic symptoms and tissue reactions, including sneezing, eye lacrimation, nose rubbing, nasal congestion, vascular congestion, inflammatory and plasma cell numbers, and eosinophil and mast cell infiltration [196]. Reduced serum IgE levels and decreased extent of goblet cell metaplasia and inflammatory cell infiltration of the lamina propria were observed [196]. In a placebo-controlled double-blind study of severe persistent allergic rhinitis adult patients, resveratrol significantly reduced nasal symptoms and improved quality of life compared to the placebo group, demonstrating reductions in IgE, IL-4, TNF-α, and eosinophil levels in the blood [197].

**Table 1:** The effect of selected small molecule natural compounds on in vitro and in vivo model systems and clinical trials, by IgE-mediated allergic disease type

Compound	Human cells/clinical trials	Animal models
*Food allergy*		
FAHF-2: 9-herbal formula: 28.17% ling zhi, 28.17% of wu mei, 1.41% chuan jiao, 8.46% huang lian, 5.63% huang bai, 8.45% gan jiang, 2.81% gui zhi, 8.45% of ren shen, and 8.45% of dang gui	*DBPCDE Phase 1*: Decrease IL-4; Safe and well-tolerated [29] *Extended Phase 1*: Safe and well-tolerated; Inhibited basophil CD63 expression ex vivo [28] *DBPC Randomized Phase 2*: Well-tolerated with no serious adverse events; No difference in epinephrine required, sIgE, IgG4, cytokine production by PBMCs or basophil activation [38] *PBMCs:* (*DPBCDE Phase 1*): Decreased IL-5 with increased IFN-ϒ and IL-10 [29] (*Phase 2*): No change in cytokine production with treatment. Baseline PBMCs decreased IL-5 and increased IL-10 and Treg cells upon treatment [38] *U266 IgE* + *Myeloma Cells:* Suppression IgE production without cytotoxicity [48, 49]	*PNA Mice*: No signs of anaphylaxis, decreased rectal temperatures or elevated histamine levels [33–35, 40, 49] IgE levels reduced and persisted after treatment; Increased IgG2A levels; Increased IFN-ϒ producing CD8 + T cell in MLN [33–35, 49] *Mouse splenocytes & MLN cells*: Decreased IL-4, IL-5 and IL-13 with increased IFN-ϒ [33–36] Reduced number peripheral blood basophils [40] *MFA Mice*: No signs of anaphylaxis, or decreased rectal temperatures or elevated histamine levels; IgE levels reduced [36]
B-FAHF-2 (BF-2), EBF-2, and E-B-FAHF-2 [Butanol- and ethyl-acetate butanol purified FAHF-2]	*U266 IgE* + *Myeloma Cells:* Suppression of IgE production without cytotoxicity [30, 48, 49] *DBPC Randomized* + *OIT* + *Omalizumab*: Safe; But outcomes not significantly improved by adding E-B-FAHF-2 [42]	*PNA Mice:* Complete protection from anaphylaxis and hypothermia [30, 44] Persistent partial protection > 50 weeks; Reduced Th2 cytokines, IgE and histamine levels [30] *PNA splenocytes and mesenteric lymph node cells*: IL-4, IL-5, IL-13 and IL-10 production reduced; IFN-ϒ was significantly increased [30] *PN-polarized SPCs:* Suppressed IL-4 and IL-5 production without cytotoxicity [30] *PNA* + *OIT:* Enhanced OIT desensitization and persistence and ameliorated adverse reaction; Lower IgE and histamine levels. Increased IFN-ϒ and IL-10 to IL-4 ratios; DNA re-methylation at IL-4 and de-methylation at IFN-ϒ and Fox-P3 promoter [39]
BBR C_20_H_18_NO_4_^+^ MW: 336.4 g/mol Isoquinoline alkaloid	*PBMCs*: Inhibited IgE production without cytotoxicity [48] IgE germline transcript expression, phosphorylated IκBα and increased T-bet, Tbx21 and STAT3 [48] *U266 IgE* + *Myeloma Cells:* Suppression of IgE production in a noncytotoxic manner [48, 49] Effects on XBP1, BLIMP1, STAT6 and rate of mitochondrial oxidation[49]	*PNA Mice:* Alone, did not reduce IgE production [44, 52] *PNA* + *BNP:* Long term tolerance with sustained reductions in IgE, symptom scores, histamine levels, body temperature reductions, and the number of IgE + B cells; Altered gut microbiota [52]
*R. cordifolia*, XPP C_14_H_8_O_4_ MW: 240.2 g/mol Anthraquinone	*U266 IgE* + *Myeloma Cells:* *R. cordifolia:* Suppression of IgE production without cytotoxicity [63] *XPP:* Suppression of IgE production without cytotoxicity [64]Altered expression of plasma cell IgE production genes, CCND1, DUSP4, SDC1, IL6R [64]	*PNA Mice*: *R. cordifolia* Reduced IgE production not IgG, anaphylaxis, hypothermia reactions, and plasma histamine levels [63] *XPP:* Reduced IgE production without affecting IgG1, IgG2a, or IgA; Anaphylaxis, hypothermia reactions, and plasma histamine levels reduced [64] High safety profile; Sustained protection 5 weeks post-treatment [64] *Murine Splenocytes & MLN*: Reduced peripheral and bone marrow IgE + B cells; Reduced IL-4 levels with increased promoter methylation without affecting IFN-ϒ [64]
A. lappa, Arctigenin C_21_H_24_O_6_ MW: 372.4 Lignin	*U266 IgE* + *Myeloma Cells:* Suppression of IgE production [76] *PNA PBMCs*: Reduced IgE, IL-5, IL-13 and percent of IgE + B cells with no effect on cell viability; Downregulation in cell division and cell-cycle genes, including NK67 and CDC45 and upregulation of anti-inflammatory genes, including BCL6 and SPARC [76]	*PNA Mice*: Reduced sIgE and histamine with no effect on IgG1 or IgA, while significantly increasing IgG1/IgG2 ratio; Blocked hypothermia and reduced symptom scores [76]
Formononetin C_16_H_12_O_4_ 268.3 g/mol Isoflavone	*U266 IgE* + *Myeloma Cells:* Suppression of IgE production by regulating the gene expression of molecular targets of IgE regulation, including NFKBIA, TP53, BCL2, BTK, STAT3, CCND1, STAT1 and NFKB1 [120] Decreased XBP1 and IgE heavy chain gene expression *FA PBMCs*: Non-cytotoxic decrease in IgE production [121]	RBL-2H3 rat basophilic cells: prevented degranulation without cytotoxicity [121], reduced histamine release and inflammatory cytokine expression [122] Mouse bone marrow derived mast cells: reduced degranulation, histamine release, and inflammatory cytokiens expression, and IgE-induced MAPK and NFκB expression [122]
Luteolin C_15_H_10_O_6_ 286.24 g/mol Flavone	N/A	*Hen Eg-White Lysozyme FA:* Prevent anaphylaxis-induced drop in blood pressure [133]
Resveratrol C_14_H_12_O_3_ 228.24 g/mol Polyphenol	Human mast cells: reduced degranulation [186] Human LAD2 mast cells: inhibited ERK phosphorylation and FcεRI- stimulated degranulation in LAD2 [187]	*OVA* + *cholera toxin- induced murine model:* Inhibits CT-driven mucosal sensitization to OVA in mice evident by reduced sIgE in serum and IL-13, and IFN-ϒ in MLN and SPL; and prevention of hypothermia [184] *Derived splenocytes (DO11.10):* Decreased OVA + CT-induced T-bet, Gata-3, IFN-ϒ, IL-4, and IL-13 [184] Inhibits early T cell activation marker CD25 + cells and IL-2 production, and co-stimulatory molecule expression in APCs CD80 and CD86 [184] *Bone Marrow Derived Dendritic Cells:* Inhibits CT-induced cAMP elevation [184] Inhibits OVA + CT-induced CD80 and CD86 expression without affecting cell viability [184] *OVA-induced Mice:* Suppressed the development of diarrhea and up-regulate the rectal temperature [185] Decreased serum IgE, MCP-1, and histamine [185] Decreased dendritic cells, B cells and mast cells in SPL and MLN [185]
*Allergic rhinitis and asthma*		
BBR C_20_H_18_NO_4_^+^ MW: 336.4 g/mol Isoquinoline alkaloid	*BEAS-2B human bronchial epithelial cells*: inhibition of IL-6 and CCL11 secretion, possibly through suppression of STAT6 signaling, without affecting cell viability [53]	*OVA-induced Rats*: Dose-dependent reductions in inflammatory cells of BALF, lung inflammation scores, NFκB signaling activity, and IgE production [57] *BBR* + *CoQ10*: Reduced nasal symptom scores, plasma IgE, IL-4, IL-13, MDA, NO levels [55] *OVA-induced Guinea Pigs*: BBR improved rhinitis symptoms including sneezing and rubbing; Decreased TNFa, IL-6, IL-1B, IL-17, and IgE in serum; Reduced IL-6, IL-1B, TLR4, PTGS2 and increased ALB mRNA expression [56] *Derf-Sensitization Mice:* *BBR*: Reduced symptom scores, sIgE, Gata-3 and Tbet and increased Foxp3 mRNA; Increased CD4 + CD25 + Foxp3 + T cells [54] *BBR (* ±*) anti-CD25 mAb*: Reduced symptom scores, serum IgE, eosinophil infiltration, increased CD4 + CD25 + Foxp3 + T cells [54] *BBR* + *anti-CD25*: serum IL-10 levels were decreased, Foxp3 mRNA decreased [54]
*R. cordifolia,* Mollugin C_17_H_16_O_4_ 284.3 g/mol Anthraquinone	*Human Jurkat T cells*: induce apoptosis through JNK and CASP-12 [67]	*ST-induced asthma murine model*: Attenuated eosinophil infiltration, epithelial mucus secretion in lung tissues, and suppressed eosinophil peroxidase activity [66] In lung tissue, lowered Th2 cytokines IL-4 and IL-5 production without affecting viability [66] Downregulated IL-4, IL-5, IL-13, eotaxin, Ccl17, Muc5ac, arginase-1, Ym-1, and Fizz-1 mRNA levels in lung tissue [66] *Peritoneal macrophages*: Reduced IL-4 stimulated increases in arginase-1 mRNA and p38 MAPK phosphorylation [66] *Mouse primary splenocytes*: Inhibited IL-4 and IL-5; Downregulated PARP1 and PAR [66]
MSSM-002 *zi su zi*, *ting li zi*, *xing ren*, *huang qin, ku sheng*, *dang gui*, *bai shao*, *ge gen*, *jie gen*, *gan cao*, *da zao*, *sheng jiang*, *zhen zhu mu*, *ling zhi*	*Human intestinal mucosal Th2- like cells* from patients with cow milk allergy: reduced proliferative responses and IL-4, IL-5, and IL-13 secretion, with no effect on IFN-ϒ production [95]	*Conalbumin-induced allergic asthma:* Eliminated AHR and pulmonary inflammation evidenced by reduced total number of cells and percentage of eosinophils in BALF; fewer inflammatory cells and no airway mucus plug in lung tissue [93] Reduced serum sIgE[93] *Splenocytes:* Reduced IL-4, IL-5 and IL-13 with no effects on IgG2A and IFN-ϒ [93] *Th2 polarized splenocytes and Th2 cloned cells (D10)*: Decreased antigen-induced proliferation without apoptosis, IL-4, IL-5 and increased IFN-Y production [94] Downregulated GATA3 mRNA and binding to IL-4 promoter [94]
ASHMI *ling zhi*, *ku sheng*, and *gen cao*	*DB Randomized:* Improved symptom scores, FEV1 values, PEF values, and reduced inhaled β2- agonist use [96] Significantly reduced numbers of peripheral eosinophils, increased serum cortisol, reduced serum IgE, IL-5, Il-13 with elevated IFN-ϒ [96] Well-tolerated, normal hematology, serum chemistry, and ECGs, with no serious adverse effects [28] *DBPCDE Randomized*: No grade 3 adverse events; Normal laboratory results; No abnormal immunological alterations- pro-inflammatory cytokines such as TNFα, IL1, and IL-6 were not increased; Safe and well-tolerated [99] *PBMCs:* Suppression of Th2 cytokines IL-5 and IL-13 without cytotoxicity [98] *U266*: Suppression of IgE [120] *Human Lung Fibroblasts:* Decreased eotaxin-1 [103] *Human Tracheal Smooth Muscle Cells (HTSMCs)*: Increased prostanoids of ASM relaxation, PGI2, without cytotoxicity [104]	*Conalbumin-induced allergic asthma:* Reduced AHR (APTI values), pulmonary inflammation (BALF total leukocyte numbers and percent eosinophils; perivascular and peribronchial inflammation grades of lung tissue), airway remodeling (PAS staining, Muc5a5 expresion, mucous cell numbers and metaplasia, PAS mRNA expression) [100] Decreased serum IgE, collage synthesis, decreased IL-4, IL-5, and IL-13 expression and increased IFN-ϒ [100] *Th2 cloned cells (D10)*: Reduced IL-4 and IL-5 [103] *Mouse pulmonary and spleen cell culture:* Decreased collagen production, sIgE and Th2 cytokines IL-4, IL-5, and IL-13, and increased IFN-ϒ [100] *Ovalbumin-induced allergic asthma:* Reduced IgE, IL-4, IL-5, IL-13 and increased IFN-ϒ, IL-10, and TGF-β [104] Sustained long-term post therapy suppression of AHR and persistent suppression of IgE, IL-4, IL-5, and IL-13 eliminated by IFN-ϒ neutralization [101, 102] Decreased mucus-positive goblet cells (PAS staining), and peribronchial and perivascular staining for collagen, eliminated by IFN-ϒ neutralization [101, 102] Decrease offspring susceptibility as maternal therapy [102] Reduced PEF, plasma histamine, LTC_4_, IgE and BALF histamine and LTC_4_; BALF percent eosinophils, lung collagen content, PAS staining, mucus goblet cells in airway [104] Reduced APTI and tracheal ring contractility upon ACh provocation, unlikely due to β2AR stimulation, but associated with PGE2 and cAMP production dependent on COX-1 activity][104–106] *Ragweed asthma murine model:* Inhibits neutrophil-predominant airway inflammation associated with TNF-α, IL-8 and IL-17 and Th1/Th2 cytokines [108] *Murine Macrophages (RAW264.7):* Reduced TNF-α production [110]
ASHMI Ganoderic Acid C1 C_30_H_42_O_7_ 514.6 g/mol Triterpenoid	*Human lung epithelial cells (NCI-H292):* Reduced Muc5A expression and ROS production in response to PMA stimulation [109] *PBMCs of Asthma Patients:* Reduced TNF-α production without cytotoxicity and inhibited p-NFκB expression [110]	*Ragweed asthma murine model:* Reduced BALF TNF-a, IL-4, IL-5 with peribronchial and perivascular inflammation in lungs [109] *Stimulated macrophages:* Reduced TNF-a production without cytotoxicity by reducing pIκB and increasing HDAC2 expression [108] Reduced total BALF cells, including neutrophils and eosinophils recruitment [109] *Murine Macrophages (RAW264.7):* Reduced LPS-stimulated TNFα production without cytotoxicity and inhibited p-IκBα, p-NFκB expression, and AP-1 and MAPK activation [110]
Formononetin C_16_H_12_O_4_ 268.3 g/mol Isoflavone	*Human epithelial bronchial cells (16HBE):* Positive effects on bronchial epithelial barrier repair including enhanced proliferation and migration with LPS-stimulation while inhibiting apoptosis via dampening Bax/Bcl-2 ratio [118] *Human nasal epithelial cells (JME/CF15)* Suppressed IL-13 upregulated IgE, histamine, TNF-α, IL-1β, IL-6, GM-CSF and eotaxin-1 proinflammatory cytokines, COX-2 and p/tNFκB expression [119] Inhibited IL-13-induced mucus formation, potentially though the upregulation of the SIRT1/Nrf2 signaling pathway [119]	*HDM-induced Asthmatic Mice:* Decreased serum levels of ALT, AST, Creatine, Uric acid, and LDH activity [118] Reduced PAS staining in lungs, levels of IgE in serum, and IL-4, IL-6, IL-10, IL-17A levels in BALF [118] Dampening Bax/Bcl-2 ratio [118]
Apigenin C_15_H_10_O_5_ 270.2 g/mol Flavone	*Jurkat T cells:* Reduction in PMA-activated increases in IL-4, IL-5, and IL-13 production without cytotoxicity [138] *Eosinophilic Leukemia Cells (EoL-1)*: Reduced butyric acid-stimulated increase in IL-5 and ECP [138] *Human lung epithelial cells (A549):* Decreased AhR and ARNT expression and enhanced AhR-silenced reductions of IL-25 and IL-33 [138] *Human bronchial epithelial cells (HBE):* Attenuated house dust mite stimulation induced reductions in ATP production and cell viability, and increase in apoptosis [139] Cytochrome c, Bax, and cleaved CASP-3 expression were downregulated, and Bcl-2 wasupregulated [139] Inhibited HDM-induced ROS production, reversed decreases in MMP and apoptosis via MAPK signaling, evident by inhibited phosphorylation of JNK, ERK, and p38 [139] *THP-1 Monocytes:* Inhibited MDC and IP-10 production and blocked phosphorylation of JNK and MAPK pathways [140] *Bronchial cells of asthma patients and human lung fibroblasts*: Attenuated TGF- β induced fibroblast-to-myofibroblast transition, in a non-cytotoxic and non-cytostatic manner; Inhibited α-SMA and tenascin C expression [141]	*OVA- Induced Asthmatic Murine Model:* Attenuated allergen-induced airway inflammation—decreased degree of inflammatory cell infiltration, airway hyperresponsiveness, total IgE levels, and demonstrating a Th1 profile [136] Inhibited airway resistance, eosinophil count, eosinophilia, leukocytosis, and lymphocytosis; Restored IgE, IL-6, TNF-α and IL-17A levels; Reduced Th17 cells [137] Reduced the expression of aryl hydrocarbon receptor in MnBP-treated; Reduced AHR, airway inflammation including total cells, macrophages, and eosinophils, peri-bronchial and peri-vascular inflammatory cell infiltration and mucus production, type 2 cytokines IL4, IL-5, and IL-13, and EPX and EPO [138] Reduced airway resistance, inflammation, mucus secretion (PAS staining) and airway remodeling (collagen volume) characterized by reductions in BALF asthma-related inflammatory cytokine including IgE, IL-4, IL-5, IL-13, and IL-17 [139] Transcriptomic alterations in pathways including MAPK signaling, Th1 and Th2 cell differentiation, and apoptosis signaling [139] Attenuates apoptosis including phosphorylation of ERKS, JNKs, and p38 MAPKs with downstream upregulation of Bcl2 and downregulation of Bax, CASP-3, and cytochrome c [139] *Splenic MNCs:* Decreased IL-4, IL-5, and IL-13 [138] *OVA-Induced Allergic Rhinitis* Reduced sIgE, IgG1, IgG2A, β -hexosaminidase, histamine, and ECP levels in mouse serum; Alleviated nasal symptoms and nasal eosinophilic infiltration and decreased Th2 cytokine and transcription factor levels, with increased Th1 cytokines and transcription factors, promoting the Th1/Th2 ratio [142]
Luteolin C_15_H_10_O_6_ 286.24 g/mol Flavone	*RAW264.7:* Interfered with HO-1 oxidative stress protein expression [161] *PBMCs of AR patients*: Reduced CD4 + IL-4 secreting cells [162] *LADR Mast Cells*: Inhibition of histamine, tryptase, MMP-9, VEGF, IL-1B, IL-6, IL-8, and TNF [167] *Human umbilical cord blood-derived culture mast cells*: inhibited the release of histamine, leukotrienes, prostaglandin D2, and GMCSF [170] *Purified basophils*: inhibited IL-4 synthesis through AP-1 transcription factor c-Jun, inhibiting AP-1 activation [170] [143]	*OVA-Induced Asthmatic Murine Model*: Modulated airway bronchoconstriction and bronchial hyperreaction with improvement in inflammation, mucus secretion and collagen deposition [158] [159] [160] Reduced OVA-specific IgE, IL-4, IL-5, and increased IFN-γ and reduced eosinophils in BALF [158, 159] [160] Reduced lung expression of eotaxin-2, CCR3, IL-13, and TNF-α and increased Foxp3, IL-10, TGF-β1, PI3K p85, p-mTOR, p-Akt, and Beclin1-PI3K3C complex [159] [160] *HDM Murine Model of Allergic Rhinitis* Prophylactic decrease in sneezing, nose scratching, mucus production, cell infiltration, eosinophils, IL-4, HDM-specific IgE, without effects on total IgE, IgG1, or IgG2a [162] Post-sensitization decreased eosinophil infiltration [162] *HDM-induced Mouse Splenocytes: Reduced* IL-4, IL-10, and IL-17 [162]
Resveratrol C_14_H_12_O_3_ 228.24 g/mol Polyphenol	*DBPC Severe Allergic Rhinitis*: Reduced nasal symptoms and improved quality of life compared to the placebo group, demonstrating reductions in IgE, IL-4, TNF-α, and eosinophil levels in the blood [197]	*HDM Murine Model of Allergic Asthma:* Attenuated pulmonary inflammation and collagen deposition with decreases in BALF IL-5, IL-17, TNF-α, and TGFβ [193] *OVA-induced Asthma Murine Model:* Reduced congestion by perivascular and perialveolar inflammatory cell infiltrate and fluid extravasation with destruction of alveolar walls [194] Decreased IL-5, IL-13 and TGFβ in BALF and serum [194] Decreased CD3 + CD4 + , CD3 + CD8 + and CD3 + CD4 + IL4 + (Th2) cells and increase in CD4 + CD25 + Foxp3 + (Treg) cells [194] Induced Foxp3 + cells in the lungs [194] *OVA-Induced Asthma Lung infiltrating cells:* Differential gene expression of anti-inflammatory pathways, including increased Foxp3 and decreased Gata-3, IL-13 and miR-34a. highlighting downregulation of miR-34a [194] *OVA-Induced Allergic Rhinitis Rat Model:* Reduced allergic symptom score, including sneezing, eye lacrimation, nose rubbing and congestion [196] Reduced vascular congestion, inflammatory and plasma cell numbers, eosinophil and mast cell infiltration, and goblet cell numbers [196] Reduced serum IgE levels [196] Decreased extent of goblet cell metaplasia and inflammatory cell infiltration of the lamina propria [196] *OVA-Induced Allergic Rhinitis Murine Model*: Reduced sneezing and nasal rubbing [195]and reduced sIgE and histamine in serum [195] Reduced sIgE, PDG2, LTC_4_, ECP, IL-4, IL-5, IL-6, IL-33, and TNF-α in NLF [195] Decreased eosinophil numbers, Txnip + cells and Txnip mRNA in nasal tissue [195] Decreased MDA levels and increased SOD activities in nasal tissue homogenates [195] *OVA-induced Allergic Rhinitis Spleen Mononuclear Cells:* Decreased Txnip protein and mRNA, MDA, ROS, IL-4, IL-5, IL-6, IL-33, and TNF-α with increased SOD activity [195]
Pterostilbene C_16_H_16_O_3_ 256.30 g/mol Polyphenol	*16HBE Cells*: Inhibition of PGE2, NO, COX-2, iNOS, IL-6, TNF-α, and IL-1β, shortened the G0/G1 phase and prolonged the G2/M phase [206] Inhibited ROS via AMPK/SIRT1 pathway [206]	*OVA-Induced Asthmatic Murine Model:* Reduced OVA-induced airway hyperreactivity, inflammatory cell infiltration and percentage of PAS + , reduction in goblet cell production and mucus secretion in lungs [206] [207] Reduced inflammatory cell number and cytokines including IL-5, IL-4, and IL-13 in BALF and total and OVA-specific IgE levels [206] Alleviated oxidative stress and airway inflammation by AMPK1/SIRT1 and NRF2/HO-1 signaling pathways [206] Decreased CD4 + IL-4 + and CD4 + -IL-13 + cells [207] *Local Passive Allergic Reaction Murine Model:* Ear tissue demonstrated reduced thickness and mast cell activation [209] *Rat Peritoneal Mast Cells*: Inhibition of degranulation and IL-6 [209] *RBL-2H3*: reduced the expression of IL-6, TNF-α, LTC4 and prostaglandin-2 through activating the LKB1/AMPK pathway and inhibits FcεRI signaling [209]
Ginsenoside Rh1 C_36_H_62_O_9_ 638.87 g/mol Steroid glycoside	*A549 Cells:* Inhibited TNF-α, IL-1β, MCP-1, ICAM-1, and MMP-9 expression and inhibition of MAPK, Akt, and NFκB activation [218] *RAW264.7 Cells:* Reduced TNF-α, IL-1β, MCP-1, iNOS, and COX2 mRNA expression [218] *HNEpCs:* increased the phosphorylation of AMPKα and reduced production of mitochondrial ROS [219]	*OVA-Induced Asthmatic Murine Model:* Total immune cell counts in the BALF, IL-4 secretion, and OVA-specific IgE reduced [218] Reduction in frequency of sneezing and nose rubbing, reduced nasal tissue thickening, submucosal eosinophils, and IL-4 + CD4 + cells, and enhancement of IFN-γ CD4 + cells [219] Reduction in serum IgE and NALF eosinophil count, and expression of IL-4, IL-5, and IL-13 [219]

Atopic dermatitis		
BBR C_20_H_18_NO_4_^+^ MW: 336.4 g/mol Isoquinoline alkaloid	Clinical S. aureus isolates: Bacteriostatic [59] Human monocytes (U937): Treated with HKSA or SA from steroid withdrawal severe eczema patients- Suppressed TNF-α; inhibited genes of AGE-RAGE and Inflammatory Signaling Pathways (IL-1β, IL-6, PTGS2, CASP3, MAPK1) and downregulated ROS without cytotoxicity [59]	NC/Nga mice: Inhibited skin severity score including itching; Reduced IgE and cutaneous infiltration of eosinophils and mast cells; Inhibited the expression of cutaneous eotaxin, MIF, and IL-4; Increased Eif3f and Malt1 [58] Murine fibroblasts: Attenuated IL-4/MIF-induced eotaxin [58] Murine macrophages (RAW264.7) + HKSA: Dose-dependent inhibition of TNF-α without cytotoxicity [59]
Formononetin C_16_H_12_O_4_ 268.3 g/mol Isoflavone	Human keratinocyte cell line (HaCaT): Reduced stimulation-induced TSLP and IL-33 expression with improved redistribution of E-cadherin [123] Suppressed TSLP production by upregulated A20 expression, through activating GPER [124] Prevents flilaggrin and loricrine expression reductions upon TNF-α/IFN-ϒ stimulation [125]	Th2-Mediated ACD Murine Model: Decreased TSLP and IL-33 levels [123] Improved ear skin epithelial cells integrity and increased E-cadherin expression [123] FITC-Induced AD Murine Model: Reduction in IgE, TSLP, and A20 [124] Increased A20 by GPER activation [124] DNCB-Induced Atopic Dermatitis Murine Model: Reduced skin lesions, swelling, ear thickness and mast cell infiltration [125]
Apigenin C_15_H_10_O_5_ 270.2 g/mol Flavone	Human keratinocytes (HaCaT): Noncytotoxic; increased genes of physical and chemical skin barrier including filaggrin, loricrin, aquaporin-3, HA and HAS-1, -2, and -3, AMPs, HBD-1, 2, -3, and cathelicidin [135]	Murine Macrophages (RAW264.7): Noncytotoxic [135] Inhibition of NO, IL-1β, IL-6, COX-2 and iNOS; Inhibited MAPK signaling molecules, including ERK, JNK and p38 [135] CD48/80-Induced Itch Mice: Reduced number of scratches and infiltration of PMNLs and mast cells [134]
Luteolin C_15_H_10_O_6_ 286.24 g/mol Flavone		RBL-2H3 Cells: Inhibition of IL-4 and degranulation [164]
Resveratrol C_14_H_12_O_3_ 228.24 g/mol Polyphenol	AD-like human keratinocytes: HA- hydrogel containing resveratrol-loaded chitosan (CS) nanoparticle counteracted oxidative damage that was induced by ROS generation without induction of cell death and time-dependently reduced the secretion and mRNA expression of proinflammatory cytokines IL-4, IL-5, IL-5, IL-13, IL-25, IL-33, and TSLP [191]	HDM + MC903 Asthma Murine Model of TSLP-Mediated Atopic March: Attenuation of airway resistance and infiltration of total cells, eosinophils, and neutrophils in ear skin [188] Dramatically less epidermal and dermal thickness of the ears [188] Ameliorates inflammatory markers TSLP, IL-4, IL-5, and IL-13 [188] Reduction in NFκB pathway related proteins p-p65, p65, p-IκBα, IκBα [188] Decreased TSLP and increased Nrf2 expression [188] AD Canine PBMCs: MCP-1 and IL-6 levels decreased with no cytotoxicity reduced [189] DNCB AD-induced mice: skin showed concentration-dependent improvement in the skin, as indicated by lesion severity score with HMGB1, RAGE, TLR4, NF-κB and pNF-κB expression reduction in mouse skin tissues and the decreased expression of pro-inflammatory cytokines TSLP, IL-4, IL-6, IL-13, IL-31 and TNF-α [190]
Pterostilbene C_16_H_16_O_3_ 256.30 g/mol Polyphenol	N/A	DNCB AD-Induced mice: Decreased epidermal thickness and decreased IgE and blood inflammatory cells and oxidative stress markers and IL-4, IL-6, TNF-α, and NF-κB in the skin [208]
Ginsenoside Rh1 C_36_H_62_O_9_ 638.87 g/mol Steroid glycoside	N/A	Oxazolone-Induced AD Mice: Reduced dryness/scaling and erosion/excoriation/hemorrhage of skin and serum IgE levels [220] Inhibited inflammatory cell infiltration in a dose-dependent manner, including mast cell infiltration and degranulation [220]

^*^ACD atopic dermatitis, *ACh* acetylcholine, *AD* atopic dermatitis, *AGE* advanced glycation end products, *AHR* airway hyperresponsiveness, *AhR* aryl hydrocarbon receptor, *AMP* antimicrobial peptides, *APC* antigen presenting cell, *APTI* peak airway pressure; *ARNT* nuclear translocator of aryl hydrocarbon receptor, *ASM* airway smooth muscle, *ATP* adenosine triphosphate, *BALF* bronchoalveolar lavage fluid, *BBR* berberine, *BNP* boiled peanut oral immunotherapy, *BTK* Bruton’s tyrosine kinase, *cAMP* cyclic adenosine monophosphate, *CASP* caspase, *CCL* CC chemokine ligand, *COX* cyclooxygenase, *CS* chitosan, *CXCL* C-X-C motif ligands, *DB* double blind, *DBPC* double blind placebo controlled, *DBPCDE* double blind placebo controlled dose escalation, *DNCB* 2,4 dinitrobenzene, *ECP* eosinophil cationic protein, *EPO* eosinophil peroxidase, *EPX* eosinophil derived neurotoxin, *ERK* extracellular signal-related kinases, *FAHF* food allergy herbal formula, *FEV1* forced expiratory volume in one second, *FITC* fluorescein isothiocyanate, *GM-CSF* granulocyte–macrophage colony-stimulating factor, *GPER* G-coupled protein estrogen receptor, *HA* hyaluronic acid, *HAS* hyaluronic acid synthase, *HBD* human β-defensin, *HDAC* histone deacetylase, *HDM* house dust mite, *HKSA* heat-killed staphylococcus aureus, *HMGB1* high mobility group box 1, *IFN* interferon, *Ig* immunoglobulin, *IL* interleukin, *JNK* c-Jun N-terminal kinases, *LPS* lipopolysaccharide, *LTC*_*4*_ leukotriene C4, *MAPK* mitogen-activated protein kinases, *MCP* monocyte chemoattractant protein, *MDA* malondialdehyde, *MDC* monocyte-derived cytokine, *MIF* macrophage migration inhibitory factor, *MIP* macrophage inflammatory protein, *MK* MAPK-activated protein kinase; *MCP1* monocyte chemoattractant protein-1, *MLN* mesenteric lymph nodes, *MMP* mitochondrial membrane potential, *MW* molecular weight, *NFκB* nuclear factor kappa-light-chain-enhanced of activated B cells, *NLF* nasal lavage fluid, *NO* nitric oxide, *NOS* nitric oxide synthase, *OIT* oral immunotherapy, *OVA* ovalbumin, *PAR* poly(ADP-ribose), *PARP* poly(ADP-ribose) polymerase, *PAS* periodic acid-schiff, *PBMC* peripheral blood mononuclear cells, *PDG2* prostaglandin D2, *PEF* peak expiratory flow, *PMA* phorbol 12-muristate 13-acetate, *PMNL* polymorphonuclear leukocytes, *PNA* peanut allergic, *PTGS2* principle isozyme responsible for production of inflammatory prostaglandins, *RAGE* receptor for advanced glycation end products, *ROS* reactive oxygen species, *SA* staphylococcus aureus, *SMA* smooth muscle actin, *SOD* superoxide dismutase, *SPC* splenocyte, *SPL* splenocytes, *ST* shrimp-tropomyosin, *STAT* signal transducers and activators of transcription, *Syk* spleen tyrosine kinase, *TGF* transforming growth factor, *Th* T helper, *TLR* toll like receptor, *TNF* tumor necrosis factor, *Treg* T regulatory, *TSLP* thymic stromal lypoprotein

### Pharmacokinetics and bioavailability

It is evident that following oral administration, resveratrol is rapidly absorbed in the gastrointestinal tract (75%) with an extensive first-pass metabolism in the intestine and liver, lowering its oral bioavailability (ranging from 2.6 to 75% in humans and 20–29% in rats fed 50 mg/kg) [198]. While 25 mg of resveratrol showed 491 ng/mL of resveratrol and its metabolites in the plasma, only trace amounts were unchanged resveratrol (< 5 ng/mL), with extremely rapid sulfate conjugation in the intestine and liver dampening resveratrol’s bioavailability [199]. This was also observed in a study that administered 500 mg of resveratrol, where much higher levels of glucoronated and sulphate resveratrol were measured compared to free, unchanged resveratrol [200]. Formulations of resveratrol have been studied for their effects on bioavailability. While large doses of resveratrol required to reach efficacy, nausea and gastrointestinal stress can occur, so a micellar 10% resveratrol solubilization formulation termed JOTROL, was investigated, and showed increasing resveratrol concentration with increased dosing [201]. Resveratrol and hydroxypropyl-β-cyclodextrin (HP-β-CD) showed great superiority in pharmacokinetic profile compared to the resveratrol and starch mixture in a human study, demonstrating 4.8 × higher resveratrol Cmax, a shorter Tmax and t1/2, and better absorption extent and rate [202]. Nanoparticle formulations have also demonstrated improvements in bioavailability [203, 204].

## Pterostilbene for IgE-mediated allergic diseases

Pterostilbene (C_16_H_16_O_3_; trans-3,5-dimethoxy-4-hydroxystilbene) is a polyphenolic compound that is the dimethylether analog of resveratrol, with two methoxy groups that increases its oral absorption and bioavailability (Figure 9A) [205]. Pterostilbene has a molecular weight of 256.30 g/mol. Its antioxidant activity has attributed to its preventative and therapeutic effects in cancer, neurological diseases, inflammation, vascular diseases, and diabetes [205]. The anti-inflammatory and antioxidant actions of pterostilbene suggested its role for managing allergic reactions.

### Asthma

Evidence that pterostilbene can reducee collagen synthesis, reticular basement membrane thickness, relieve hyperplasia, and airway remodeling implicated it as an allergic asthma treatment option [206]. Mice with OVA-induced asthma were treated with 30 or 50 mg/kg pterostilbene and lungs were investigated for airway inflammation [206]. Pterostilbene treatment significantly reduced OVA-induced inflammatory cell infiltration and percentage of PAS+, indicating reduction in goblet cell production and mucus secretion (Figure 9B, C) [206]. The BALF showed dose-dependent reduction in inflammatory cell number and cytokines including IL-5, IL-4, and IL-13, in a dose -dependent manner [206]. Total and OVA-specific IgE were also decreased upon treatment (Figure 9D). Airway hyperreactivity was also diminished with pterostilbene treatments [206]. In vitro, 16HBE cells when treated up to 100 μM of pterostilbene showed no cytotoxicity, and a dose-dependent inhibition of PGE2, NO, COX-2, and iNOS were observed compared to untreated groups, upon LPS stimulation [206]. Inhibition of IL-6, TNF-α, and IL-1β were also observed. While LPS stimulation increases the G0/G1 phase and decreases the G2/M phase, pterostilbene treatment shortened the G0/G1 phase and prolonged the G2/M phase. In vivo, oxidative stress as indicated by serum SOD, CAT, and MDA, showed increase in SOD and CAT levels and decrease in MDA upon treatment [206]. In vitro, 16HBE cells expression of ROS was inhibited with pterostilbene treatment [206]. Treatment increased p- AMP-activated protein kinase (AMPK) and SIRT1 expression in the asthmatic mouse lung and stimulated 16HBE cells, suggesting the activation of the AMPK/SIRT1 pathway [206]. Further, in both mouse lung tissue and 16HBE cells, NRF2 and HO-1 protein expression were increased [206]. Oxidative stress and airway inflammation were therefore alleviated by pterostilbene via the AMPK1/SIRT1 and NRF2/HO-1 signaling pathways in allergic asthma. In another HDM- asthmatic mouse model, pterostilbene was administered intraperitonially at 10, 20, or 40 mg/kg/day for 4 days as a prophylactic measure before inducing asthma [207]. Pterostilbene prophylactic significantly attenuated airway hyperresponsiveness and dose dependently inhibited CD4+IL-4+T cells with no effect on lymph node CD4+IL-17A+or CD4+IFN-γ+T cells [207]. The determined optimal dose, 20 mg/kg, decreased serum levels of IL-13, total IgE, and total IgG, with no effect on IL-5 or IL-10 [207]. Pterostilbene reduced infiltration of eosinophils in the lungs without affecting monocytes or mucus production [207]. When investigated as a therapeutic following induction of asthma, pterostilbene significantly ameliorated disease severity, explained by the decrease in elevation of lymph node, splenic and pulmonary CD4+IL-4+T cells, decrease in serum IL-13, IgE and IgG levels, and reduction in pulmonary eosinophil infiltration [207]. Th2 CD4+ cells were used to determine the effect of pterostilbene (10 μM) on Th2 polarization, and a decrease in CD4+IL-4+ and CD4+IL-13+ cell populations were observed [207]. Treatment decreased glycolysis without affecting oxidative phosphorylation of the Th2 cells, significantly decreasing the levels of pyruvate and acetyl-CoA [207]. The expression of GATA3 was also suppressed and agreed with downregulated mTOR and Glut1 levels [207]. H3 histone acetylation, IL-4 and IL-13 were also reduced [207]. In addition, eosinophil H4 histone deacetylation, differentiation, and IL-4 production were decreased, highlighting the regulation of glycolysis-directed Th2 cell proliferation, maturation and epigenetic changes via mTOR pathways by pterostilbene [207]. Taken further, Th2 cells from asthmatic human patients’ PBMCs treated with pterostilbene in vitro, saw inhibited CD4+IL-4+ cells with HDM stimulation [207].

### Atopic dermatitis

To identify the effects of pterostilbene on atopic dermatitis, topical pterostilbene (0.2, 0.6, and 1% w/w) was applied to DNCB sensitized mice [208]. Reduced ear weight, skin thickness, and the weight and size of thymus glands and spleen were observed [208]. Pterostilbene treatment decreased atopic dermatitis- induced epidermal thickness and decreased IgE and blood inflammatory cells. In the skin, oxidative stress markers and IL-4, IL-6, TNF-α, and NF-κB were also reduced [208]. Authors urged for further investigation of pterostilbene in atopic dermatitis and other inflammatory skin disorders.

### Mast cells

Pterostilbene was also investigated for its effects on mast cell degranulation in vitro and in vivo [209]. Mast cell-mediated local passive allergic reactions in mice were assessed following treatment with 10 mg/kg or 20 mg/kg pterostilbene [209]. Ear tissue demonstrated reduced thickness and mast cell activation induced by antigen following pterostilbene treatment [209]. Rat peritoneal mast cells were also treated with pterostilbene (1, 10, 50, 100 μM) and no effect on cell viability was observed, however degranulation and IL-6 expression was inhibited [209]. Pterostilbene treatment of 15 and 30 μM showed reduced histamine secretion and calcium uptake, overall indicating its ability to prevent degranulation, histamine release, and intracellular calcium level of mast cells [209]. Similarly, pterostilbene treatment was not cytotoxic to RBL-2H3 cells, yet reduced the expression of IL-6, TNF-α, LTC_4_ and prostaglandin-2 at 15 and 30 μM [209]. Mechanistically, pterostilbene treatment of RBL-2H3 cells increased phosphorylation of Serine/Threonine Kinase 11 (LKB1), AMPK, and acetyl co-A carboxylase, and decreased proteins involved in FcεRI-mediated signaling, including phosphorylated p38, ERK, JNK, IKK and mTOR, suggesting pterostilbene both activates the LKB1/AMPK pathway and inhibits FcεRI signaling [209]. This highlights the use of pterostilbene as a therapeutic against mast cell-mediated allergic diseases.

### Pharmacokinetics and bioavailability

Pterostilbene has had its pharmacokinetic profile directly compared to resveratrol, exhibiting superiority (3-4x), due to its dimethyl ether structure increasing metabolic stability and bioavailability (66.9% vs. 29.8%) [198, 210]. Pterostilbene (56 mg/kg) demonstrates 36× higher peak plasma concentration compared to resveratrol (50 mg/kg) [210]. Pterostilbene (28 mg/kg) has been found to be distributed across various tissues, notably able to pass the blood–brain barrier [211]. It has been demonstrated that,opposed to resveratrol’s 68% unmodified, pterostilbene at over 75% remains unchanged following metabolism [212]. Additionally, pterostilbene has a limited elimination ability and has a lower clearance rate than resveratrol [213]. Yeo et al., also demonstrated that fasting status impacts pterostilbene bioavailability in addition to its poor aqueous solubility [213]. Demonstrating poor water solubility that affects its absorption, methods have been used to improve it, including co-crystallization with piperazine and 2-hydroxypropyl-β-cyclodextrin complex [213, 214].

## Ginsenoside Rh1 for IgE-mediated allergic diseases

Ginsenosides are the major bioactive components of *Panax ginseng*, demonstrating pharmacological activities including anti-cancer [215, 216] and anti-inflammation [217]. Ginsenoside Rh1 (C_36_H_62_O_9_) is a steroid glycoside (saponin) with a molecular weight of 638.9 g/mol that has been investigated for its anti-allergic properties (Figure 10A).

### Asthma and allergic rhinitis

Identification of novel therapeutics from natural sources for asthma progression enabled the investigation of ginsenoside Rh1 [218]. Ginsenoside Rh1pre-treatment of A549 cells prevented 50 nM PMA stimulation-induced TNF-α, IL-1β, and MCP-1 mRNA expression in a dose-dependent manner (5, 10, 25, 50 μM) [218]. PMA-induced expressions of ICAM-1 and MMP-9 at the RNA and protein level in a dose-dependent manner were also observed[218]. At 25 and 50 μM, in a dose-dependent manner, Ginsenoside Rh1 significantly reduced expression of p-ERK1/2, p-JNK, p-p38, pAkt, and p-NFκB-p65, confirming inhibition of MAPK, Akt, and NFκB activation [218]. Specifically, nuclear translocation of NFκB was significantly inhibited at 50 μM of ginsenoside Rh1 pretreatment [218]. Murine RAW264.7 macrophages were induced with LPS following pre-treatment with Ginsenoside Rh1 at 5, 10, 25, and 50 μM, and confirmed that at as low as 5 μM, TNF-α, IL-1β, MCP-1, iNOS, and COX2 mRNA expression were reduced. At 25 and 50 μM of Ginsenoside Rh1 pretreatment, p- NFκB -p65 and iNOS protein levels were decreased. In their in vivo model of OVA/LPS-induced allergic asthma, mice were treated with 20 or 40 mg/kg of ginsenoside Rh1[218]. Total immune cell counts in the BALF were significantly reduced, including infiltrated macrophages, neutrophils, and eosinophils upon treatment [218]. Further, IL-4 secretion and OVA-specific IgE production were significantly reduced by ginsenoside Rh1 treatment, demonstrating its protection against allergic asthma by suppression of immune cell infiltration and activation of MAPK, Akt, and NFκB signaling [218].

Preclinical in vivo and in vitro models were used to ascertain if ginsenoside Rh1 alleviated allergic rhinitis symptoms via mitochondrial autophagy [219]. In their HDM mouse model, ginsenoside Rh1 treatment, notably at 50 mg/kg, caused a reduction inf frequency of sneezing and nose rubbing (Figure 10C), which coincided with substantial reduction of nasal tissue thickening, submucosal eosinophils, and IL-4+CD4+ cells, and enhancement of IFN-γ CD4+ cells [219]. Serum IgE and NALF eosinophil count and expression of IL-4, IL-5, and IL-13 were also reduced, supporting a Th1/Th2 balance correction by ginsenoside Rh1 treatment (Figure 10B) [219]. Nasal mucosal epithelial cells demonstrated reduced apoptotic marker expression including cleaved-CASP-3, cyt-c, NLRP3, ASC, IL-1β, IL-18, CASP-1, and cleaved CASP-1, and increased Bcl-2, confirmed by TUNEL-positive apoptotic cell reduction, suggesting an inhibition of anti-apoptotic and anti-inflammatory effects on nasal tissue [219]. In silico analysis elucidated the AMPK/ULK1/FUNDC1 pathway, and upregulation of p-AMPK, p-ULK1, and p-FUNDC1 expression was confirmed in the nasal mucosa [219]. In vitro, HNEpCs cells treated with 50 μM of ginsenoside Rh1 had significantly increased the phosphorylation of AMPKα and reduced production of mitochondrial ROS, indicating that Rh1 may influence mitochondrial autophagy [219]. In all, ginsenoside Rh1 protects from allergic rhinitis through reducing inflammatory responses and inhibiting apoptosis, mediated by the AMPK/ULK1/FUNDC1 mitophagy pathway.

### Atopic dermatitis

Atopic dermatitis alleviation by ginsenoside Rh1 was investigated in an oxazolone-induced atopic dermatitis-like skin lesion model via oral administration at 20 mg/kg or 10 mg/kg [220]. Ginsenoside Rh1 improved skin symptoms at both doses, including reduced dryness/scaling and erosion/excoriation/hemorrhage [220]. Additionally, ear swelling and weight were significantly suppressed [220]. Dorsal skin showed inhibition of inflammatory cell infiltration in a dose-dependent manner, including mast cell infiltration and degranulation [220]. This coincided with reduced serum IgE levels in both groups, and significantly reduced IL-6 levels when treated with 20 mg/kg [220]. Ginsenoside Rh1 at 20 mg/kg did not decrease mRNA expression of IL-4, but increased IFN-γ and Foxp3 expression [220].

### Pharmacokinetics and bioavailability

Following oral administration of ginseng extract, ginsenoside Rh1 undergoes hydrolysis and metabolism in the gastrointestinal tract, with demonstrated absence in plasma [221, 222]. Ginsenoside Rh1 bioavailability was 1.01% in one study, highlighting pre-systemic metabolism as a partial explanation [223]. Fast absorption of ginsenoside Rh1 has also been reported [224]. Ginsenoside Rh1 also serves as a metabolite for ginsenoside Rg1 and ginsenoside Re [225]. Due to the biotransformation of ginsenosides and low bioavailability, the full pharmacokinetic profile of pure ginsenoside Rh1 is limited and warrants further study. Addressing its poor solubility, rapid metabolism, and low membrane permeability through delivery systems including nanocarriers may prove beneficial, but research is limited. Rh1-loaded vesicular systems such as liposomes, ethosomes, and transfersomes have also been investigated and showed higher skin permeation [226]. Elucidation of the ginsenoside Rh1 pharmacokinetic profile and improved delivery systems warrants further investigation.

## Discussion

While the herbal formulations and their active compounds, standalone or in combination, have demonstrated robust in vitro efficacy, translation to in vivo and clinical settings remains variable, and oftentimes a large therapeutic barrier. The concentrations achieved in vitro evidently exceeds what is achieved with oral administration, particularly for compounds with limited bioavailability, extensive metabolism, and broad tissue distribution. Pharmacokinetic profiles reveal low plasma levels and rapid clearance, confirming the unattainable therapeutic dose using pure compounds or conventional formulations, even with synergistic effects. While constraining, formulation strategies to enhance solubility, absorption, and systemic exposure have demonstrated rational and increasingly validated means to bridge the gap seen between mechanistic efficacy and clinical translation.

## Conclusion

Definitive conclusions regarding natural compounds remain challenging due to variations in formulation, routes of administration, and the limited size of clinical studies. Nonetheless, ongoing research seeks to clarify their mechanisms of action and safety, which may yield effective natural products for allergic diseases mediated by pathological IgE. Importantly, novel herbal compounds have progressed through vigorous stages of refinement, including formulation, purification, and identification of the active ingredients that specifically modulate IgE-mediated responses. These compounds show promise as standalone therapies and in combination with established treatments, as harnessing their anti-inflammatory actions can be broad, yet targeted, suppressing multiple cytokines simultaneously (Table 1, Fig. 8). Their favorable safety profile, extended into infants, supports early clinical application. Given their potential to provide effective, affordable, and well-tolerated options to allergy sufferers, naturally derived small molecule compounds have become compelling for investigation. Encouragingly, great benefits have been demonstrated, including symptom control and increased quality of life, for patients with IgE-mediated diseases including food allergy, allergic rhinitis, asthma, and eczema.

## Data Availability

Data sharing is not applicable to this article as no datasets were generated or analyzed during the current study.
